# Upregulating *KTN1* promotes Hepatocellular Carcinoma progression

**DOI:** 10.7150/jca.55570

**Published:** 2021-06-11

**Authors:** Jian Pan, Nai-Xia Chao, Yao-Yao Zhang, Tian-Ming Huang, Cheng-Xiao Chen, Qiu-Hong Qin, Jin-Hong Guo, Rong-Shi Huang, Guo-Rong Luo

**Affiliations:** 1 Department of Human Anatomy, Guangxi Medical University.; 2 Department of Biochemistry and Molecular Biology, Guangxi Medical University.; 3 Department of Histology and Embryology, Guangxi Medical University.; 4 Department of Histology and Embryology, Guangxi Medical University.; 5 The Ninth Affiliated Hospital of Guangxi Medical University, Guangxi Medical University.; 6 Jiang bin Hospital of Guangxi Zhuang Autonomous Region.; 7 Guangxi Medical University.; 8 Department of Histology and Embryology, Guangxi Traditional Chinese Medical University.; 9 Department of Histology and Embryology, Guangxi Medical University.; 10 Guangxi Colleges and Universities Key Laboratory of Human Development and Disease Research, Guangxi Medical University, Nanning, China.

**Keywords:** hepatocellular carcinoma, kinectin 1, CRISPR/Cas9, mechanism

## Abstract

**Background**: Hepatocellular carcinoma (HCC) presents a common malignant tumor worldwide. Although kinectin 1 (*KTN1*) is the most frequently identified antigen in HCC tissues, the detailed roles of *KTN1* in HCC remain unknown. This study seeks to clarify the expression status and clinical value of *KTN1* in HCC and to explore the complicated biological functions of *KTN1* and its underlying mechanisms.

**Methods**: In-house reverse transcription quantitative polymerase chain reaction (RT-qPCR) was used to detect the expression of *KTN1* in HCC tissues. External gene microarrays and RNA-sequencing datasets were downloaded to confirm the expression patterns of *KTN1*. The prognostic ability of *KTN1* in HCC was assessed by a Kaplan-Meier curve and a hazard ratio forest plot. The CRISPR/Cas9 gene-editing system was used to knock out *KTN1* in Huh7 cells, which was verified by PCR-Sanger sequencing and western blotting. Assays of cell migration, invasion, viability, cell cycle, and apoptosis were conducted to explore the biological functions. RNA sequencing was performed to quantitatively analyze the functional deregulation in *KTN1*-knockout cells compared to Huh7-wild-type cells. Upregulated genes that co-expressed with *KTN1* were identified from HCC tissues and were functionally annotated.

**Results**: *KTN1* expression was increased in HCC tissues (standardized mean difference [SMD] = 0.20 [0.04, 0.37]). High *KTN1* expression was significantly correlated with poorer prognosis of HCC patients, and *KTN1* may be an independent risk factor for HCC (pooled HRs = 1.31 [1.05, 1.64]). After *KTN1*-knockout, the viability, migration, and invasion ability of HCC cells were inhibited. The proportion of HCC cells in the G0-G1 phases increased after *KTN1* knockout, which also elevated the apoptosis rates in HCC cells. Several cascades, including innate immune response, chemical carcinogenesis, and positive regulation of transcription by RNA polymerase II, were dramatically changed after *KTN1* knockout. *KTN1* primarily participated in the cell cycle, DNA replication, and microRNAs in cancer pathways in HCC tissues.

**Conclusion**: Upregulation of *KTN1* served as a promising prognosticator in HCC patients. *KTN1* promotes the occurrence and deterioration of HCC by mediating cell survival, migration, invasion, cell cycle activation, and apoptotic inhibition. *KTN1* may be a therapeutic target in HCC patients.

## Introduction

Hepatocellular carcinoma (HCC) is one of the most concealed, malignant, lethal solid tumors in the world. Nearly seven million people are diagnosed with HCC and over six million people die from it per year 1, 2. It predominates in Africa and eastern Asia, especially China, where Guangxi possesses large numbers of HCC patients 3, 4. Chronic hepatic diseases, metabolic syndromes, and smoking are primary risk factors for HCC 5-7. Although the exact pathogenesis of HCC remains unclear, its development involves successive stages of inflammation, pathogenic genetic alterations, and epigenic deregulation 8-13. Surgical resection is the optimal choice for HCC patients in addition to transplantation, chemoembolization, and local ablation 14-17. However, many HCC patients lack access to this treatment opportunity because of the absence of symptoms in the early stages and progression in late HCC 18-20. Furthermore, HCC patients in the same clinical stages have inexplicably distinct outcomes. A classification of molecular subtypes was proposed to stratify HCC patients according to molecular signatures, such as TP53 mutation and the AKT-mammalian target of rapamycin activation 21. Nonetheless, more studies are necessary to lay the foundations of HCC treatment and prognosis improvement.

Kinectin 1, encoded by *KTN1* (also called *KNT*, *CG1*, and *MU-RMS-40.19*), belongs to an integral membrane protein in the endoplasmic reticulum and participates in organelle transportation 22. The *KTN1* gene is renowned for its relationships with axon transport and cell mitosis 23, and the KTN1 protein regulates the expression of other proteins by binding to translational elongation factor (EF)-δ and participating in the assembly of the EF-1 complex 24, 25. Studies have confirmed several roles of *KTN1* in certain cancers. For instance, *KTN1* was significantly upregulated in cutaneous squamous cell carcinoma (CSCC), and a *MALAT1*-*KTN1*-*EGFR* axis, which also existed in melanoma and cervical cancer cells, was demonstrated to promote the development of CSCC cells 26. *KTN1* expression also significantly increased in giant cell tumors of bone compared to normal controls 27. In HCC, researchers studied HCC-related antigens to determine their clinical implications and reported 24 antigens, where *KTN1* was the most common 23. Moreover, researchers have discovered four alternative splicing sites of *KTN1* in HCC: D1 and D4 were responsible for the regulation of protein and mRNA expression, while D2 and D3 mainly affected the molecular function of protein. However, the relationships between *KTN1* and HCC have not yet been studied, and the deregulation level of *KTN1* and its biological effects in HCC are largely unknown, which must be clarified to understand the roles of *KTN1* in HCC.

Therefore, this study clarifies the expression status and clinical value of *KTN1* in HCC. More importantly, this study explores the complicated biological functions of *KTN1* in HCC cells to pinpoint its underlying mechanisms.

## Materials and methods

### Expression status and clinical value of *KTN1* in HCC

#### RT-qPCR detection of KTN1 expression in HCC tissues

A total of 50 pairs of surgically resected HCC and adjacent tissues were collected from the First Affiliated Hospital of Guangxi Medical University. The HCC patients had to meet the following inclusion criteria: i) pathological diagnosis was HCC and ii) they signed an informed consent form. This study was approved by the ethics committee of the First Affiliated Hospital of Guangxi Medical University. Total RNA was extracted using the AxyPrep Multisource Total RNA Miniprep Kit (AXYGEN) and was reversely transcribed into cDNA using the HiScript III RT SuperMix for quantitative polymerase chain reaction (qPCR) (Vazyme). A 2X Universal SYBR Green Fast qPCR Mix (ABclonal) reagent was used to perform real-time reverse transcription quantitative polymerase chain reaction (RT-qPCR). The primers were as follows: *KTN1* primer upstream: 5'-GGCAGAGATAGCTCACTTGAAG-3'; downstream: 5'-ACCTAGCATAATCCTGGCGTAG-3' (125bp); internal control (Actin Beta) primer upstream: 5'-CAGGCACCAGGGCGTGAT-3'; downstream: 5'-TAGCAACGTACATGGCTGGG-3' (293bp). A LightCycler96 was used according to the protocols of the qPCR reagents. The reaction system was as follows: 10 μl of mix, 0.3 μM of upstream primers, 0.3 μM of downstream primers, and 1 μl of cDNA template, and then add water for a total volume of 20 μl. The reaction conditions contained 95 °C for 3 minutes pre-denaturation, 95 °C for 15 seconds, and 60 °C for one minute. The latter two processes were looped 40 times. The Ct values of the internal control and *KTN1* were collected, and the relative expression of *KTN1* was calculated by the 2^-ΔΔCt^ method.

#### External validation of KTN1 expression by gene microarrays and RNA-sequencing datasets

Gene microarrays and RNA-sequencing (RNA-seq) datasets were downloaded from the public biomedical databases Gene Expression Omnibus, Oncomine, ArrayExpress, the Cancer Genome Atlas, and Genotype-Tissue Expression. Three authors independently screened these studies according to the following criteria: i) the organism was a human being, ii) the specimen type was tissue, and iii) the tumor was HCC. Datasets or samples were excluded using the following criteria: i) the mRNA expression level of *KTN1* was not detected or ii) the samples were duplicated. Finally, the three authors discussed the screened datasets and reached an agreement. The enrolled datasets were merged into platform matrices using dplyr, and the batch effect was removed using sva. The expression values of *KTN1* were extracted from the batch-effect-removed platform matrices. T-tests and SMDs were used to determine the expression levels of *KTN1* in HCC.

#### Potential clinical value of KTN1 in HCC patients

Summary receiver operating characteristic (SROC) curves, sensitivity and specificity forest plots, and positive and negative likelihood ratio forest plots were utilized to appraise the discriminatory ability of *KTN1* between HCC and normal liver tissues. HCC patients were divided into *KTN1* high- and *KTN1* low-expressing groups. Kaplan-Meier curves with a log-rank test and pooled hazard ratios (HRs) forest plots were used to appraise the prognostic value of *KTN1* in HCC patients. To explore the potential roles of *KTN1* in HCC progression, Barcelona Clinic Liver Cancer (BCLC) and tumor, node, metastases (TNM) staging subgroups were set up, and the expression patterns of *KTN1* in progressed HCC were addressed. Three gene chips (i.e., GSE63018, GSE40367, and GSE364) were used to analyze the possible roles of *KTN1* in HCC metastasis.

### Biological functions of *KTN1* in HCC cells

#### Cell culture

The human cell line Huh7 was maintained at 37 °C in a constant-temperature incubator with 5% CO_2_. Cells were grown in Dulbecco's Modified Eagle Medium (DMEM) medium (Gilbco) with 10% fetal bovine serum (Gilbco).

#### Construction of CRISPR/Cas9 plasmid

The pSpCas9(BB)-2A-Puro (PX459) v2.0 (Plasmid #62988) plasmid came from the Addgene website. The plasmid was constructed according to the protocols provided by the website (https://media.addgene.org/data/plasmids/62/62988/62988-attachment_i-jdFt6Gm-ft.pdf). The gRNA sequence was 5'-GTTTCCTCTTCTTCTGGCTTTTC-3'.

#### Knockout of KTN1 in Huh7 cells by using CRISPR/Cas9

Clustered regularly interspaced short palindromic repeats/CRISPR-associated protein 9 was used to knock out *KTN1*. Each six-well plate was seeded with 5×10^5^ cells. After 24 hours, the cells were transfected with lipo3000 according to the instructions of the production company (https://www.thermofisher.com/order/catalog/product/L3000150?SID=srch-srp-L3000150). Two μg of plasmid and 4 μl of P3000 were added to 100 μl of reduced-serum media (opti-MEM) (Gilbco) and mixed gently. Then, 100 μl of opti-MEM and 6 μl of lipo3000 were added to another eppendorf tubes, mixed gently, and stood for five minutes. The diluted plasmid was added to the diluted lipo3000. After standing at room temperature for 20 minutes, the mixed liquid was added to the cells. After 24 hours of transfection, it was changed to a complete medium containing 2 μg/ml puromycin for 48 hours and then changed back to a complete medium without puromycin to continue the culture.

#### Efficiency verification of CRISPR/Cas9-mediated KTN1 knockout

To verify the knockout effect at the DNA level, cell genomic DNA was extracted using the phenol-chloroform method. Primers on both sides of the knockout site were designed, and PCR amplification was performed. Sanger sequencing was conducted to identify the knockout effect. The sequence of the PCR primers was as follows: upstream primer: 5'-TTTCCTCTTCTTCTGGCTTTTC-3'; downstream primer: 5'-AACGACATTCAATGGAACAGGT-3' (253 bp).

To verify the knockout effect at the protein level, western blotting was performed. Cell lysate (94 μL cell lysate plus 6 μL PMSF) was added to the collected 1×10^6^ cells, which was operated on ice. The ice box was placed on a shaker and shaken for 30 minutes, followed by 12,000-rpm centrifugation at 4 °C for 20 minutes. The protein concentration was determined by the bicinchoninic acid assay. Protein loading buffer was added to 20 μg of protein and boiled for 10 minutes. After that, sodium dodecyl sulphate-polyacrylamide gel electrophoresis was performed, where the concentration of concentrated gel was 5%, the concentration of separation gel was 10%, the voltage was 90 V, and the electrophoresis time was 90 minutes. Then, the protein was transferred to the polyvinylidene difluoride (PVDF) membrane, which was sealed in 5% skim milk at room temperature for 1 hour. The primary antibody, rabbit anti-*KTN1* polyclonal antibody (Abcam), was diluted at 1:1000 with Tris-buffered saline with Tween-20 (TBST). The PVDF membrane was incubated in the primary antibody at 4 °C overnight and was washed three times with TBST for 5 minutes each time. The PVDF membrane was then incubated with the secondary antibody, goat anti-rabbit IgG H&L (Abcam), which was diluted at 1:10000 with TBST and incubated at room temperature for 2 hours. After washing the membrane three times with TBST again, imaging was performed using the ChemiDoc™ MP Imaging System (Bio-Rad).

#### Cell viability assay

In total, 2,000 cells were seeded in each well of a 96-well plate. After 24 hours, a CellTiter-Glo Luminescent Cell Viability Assay (Promega) kit was used to detect cell viability and draw cell growth curves.

#### Cell migration assay

A 24-well plate transwell chamber (Corning, Costar 3422) was used to test the cell migration ability. Respectively, 100 μl and 500 μl of the medium containing 0.5×10^5^ cells were added to the upper and lower chambers. The medium in both chambers was DMEM containing 5% FBS. After incubation at 37 °C for 24 hours, the chambers were removed, and the cells on the top surface of the chambers were gently wiped using a cotton swab. The chambers were washed twice with phosphate buffer saline (PBS) and then fixed in methanol for 30 minutes. When the chambers were dry, 0.1% crystal violet was used to stain for 30 minutes, and excess crystal violet was washed off using PBS. The cells were observed under a microscope after drying.

#### Cell invasion assay

The cell migration ability was tested by using a 24-well plate transwell chamber (Corning, Costar 3422). Matrigel (BD, 356234) was diluted with serum-free DMEM (Matrigel: DMEM = 1:8), and 60 μl of it was added to the chamber and incubated at 37 °C for 1 hour. After aspirating the liquid, the chamber was washed twice using DMEM. Then, 100 μl of serum-free DMEM containing 0.5×10^5^ cells was added to the upper chamber, and 500 μl of DMEM containing 5% FBS was added to the lower chamber. The transwell chamber was incubated at 37 °C for 24 hours. The subsequent steps were the same as the detection of cell migration above.

#### Cell cycle assay

In total, 5×10^5^ cells were centrifuged at 1,200 rpm for 5 minutes, and the supernatant was removed. One ml of pre-cooled 75% ethanol was added to the remaining component, and after resuspending, the cells were fixed at 4 °C for 2.5 hours. After centrifuging at 1,200 rpm for 5 minutes, the supernatant was discarded. One ml of PBS was used to wash the cells once, which were centrifuged again and had their supernatant discarded. Propidium Iodide/ribonuclease dye was used for staining. The cells were incubated in the dark at room temperature for 15 minutes, and flow cytometry was used to perform detection within 0.5 hours.

#### Cell apoptosis assay

In total, 10^5^ cells were centrifuged at 1,200 rpm for 5 minutes, and the supernatant was removed. Then, 50 μl of 1× binding buffer was added to each tube, and after resuspending, different dyes were added according to the groups as follows: I) negative control: no dye; II) single positive control: 5 μl of Annexin V-APC; III) single positive control: 10 μl of 7-AAD. Five μl of Annexin V-FITC and 10 μl PI were added to the sample tube. After shaking the solution gently, it was incubated at room temperature (25 °C) in the dark for 15 minutes, and then 200 μl of 1× binding buffer was added. Flow cytometry was performed for detection within 1 hour.

### RNA-seq

#### Total RNA extraction and RNA-seq

The total RNA extracted from the samples was treated with DNase I, and the magnetic beads with Oligo (dT) were used to enrich the eukaryotic mRNA. A breaking reagent was added to break the mRNA into short fragments, and a strand of cDNA was synthesized with six-base random primers (random hexamers) using the fragmented mRNA as a template. Buffer, dNTPs, and DNA polymerase I were added to synthesize the second strand of cDNA, which was purified and recovered by the kit. After being repaired by sticky ends, added with base “A” at the 3' end, and connected with a sequencing adapter, the resulting fragments were subjected to size selection, PCR amplification, and enrichment. The constructed library was qualified by an Agilent 2100 Bioanalyzer and Applied Biosystems StepOnePlus RT-PCR System. The Illumina sequencing platform was used for sequencing.

#### Data analysis of RNA-seq

The raw reads obtained by the sequencing platform were subjected to data quality control and filtered to obtain high-quality clean reads, which were aligned to the reference genome. The reads of the aligned genome were further located to the gene exon region using HTseq v0.6.1, and the number of reads on each gene alignment was counted to estimate the gene expression level. To make the estimated gene expression levels of different genes and different experiments comparable, the fragments per kilobase per million fragments (FPKM) 28 were calculated according to the following formula:


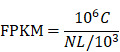


where C is the fragments (pair-end reads) uniquely aligned to the gene, N is the total number of fragments (pair-end reads) uniquely aligned to the reference gene, and L is the number of bases in the coding region of the gene.

#### Differentially expressed gene analysis, gene ontology (GO), WEGO, and pathway enrichment

Gene-expression analysis was conducted to learn the possible mechanisms of *KTN1* in HCC cells. Significantly differentially expressed genes (DEGs) in *KTN1*-KO cells, compared to Huh7-WT cells, were identified using DEGseq v1.18.0 and DEGseq2 v1.4.5. The screening conditions were as follows: I) fold change ≥2 and II) Q-value ≤0.01. Gene ontology (GO) analysis 29 was performed using GOseq v1.16.2 and topGO v2.16.0. (Web Gene Ontology) WEGO software was used to compare the GO entries of the DEGs with the GO entries of all the reference genes and to find the GO terms that were significantly different from the background. A Kyoto Encyclopedia of Genes and Genomes (KEGG) 30 analysis was also conducted.

#### Risk prediction signatures based on Cox regression model

Protein-protein-interaction (PPI) analysis was performed to confirm the intimate interaction between *KTN1* and DEGs, which was identified from in-house RNA-seq analysis. A PPI network was constructed using the networkD3 package. Hub genes were analyzed. A Cox regression model was utilized to determine putative risk indicators for HCC patients. HCC patients from The Cancer Genome Atlas (TCGA) cohort were grouped into high- and low-risk groups according to the median log (CPM+1) expression value of the putative risk indicators. Ggrisk and rms packages were exploited to analyze the risk distribution and survival conditions between the groups. The two-year and five-year survival rates of HCC patients were predicted based on these gene panels. Calibration curves were drawn and a C-index calculated to assess the accuracy of the established prognostic gene signatures.

### Co-expression regulatory network analysis

To explore the potential mechanisms of *KTN1* underlying HCC tissues, functional enrichment was performed based on gene expression analysis by using the integrated external gene chips and RNA-seq datasets. DEGs in HCC tissues, compared to normal liver tissues, were identified by using the limma-voom package. The filtration conditions were as follows: I) log_2_ fold change ≥1 and II) adjusted P <0.05. Co-expressed genes (CEGs) positively related to *KTN1* were identified by performing gene co-expression analysis. The criteria were as follows: I) Person's relation coefficient >0.3 and II) P < 0.05. DEGs were intersected by CEGs. GO, KEGG, disease ontology (DO) 31, WikiPathways 32, and Reactome pathways 33 were enriched according to the overlapping genes. Finally, a PPI network was constructed.

## Results

### Increased *KTN1* expression in HCC tissue

**Figure 1:**
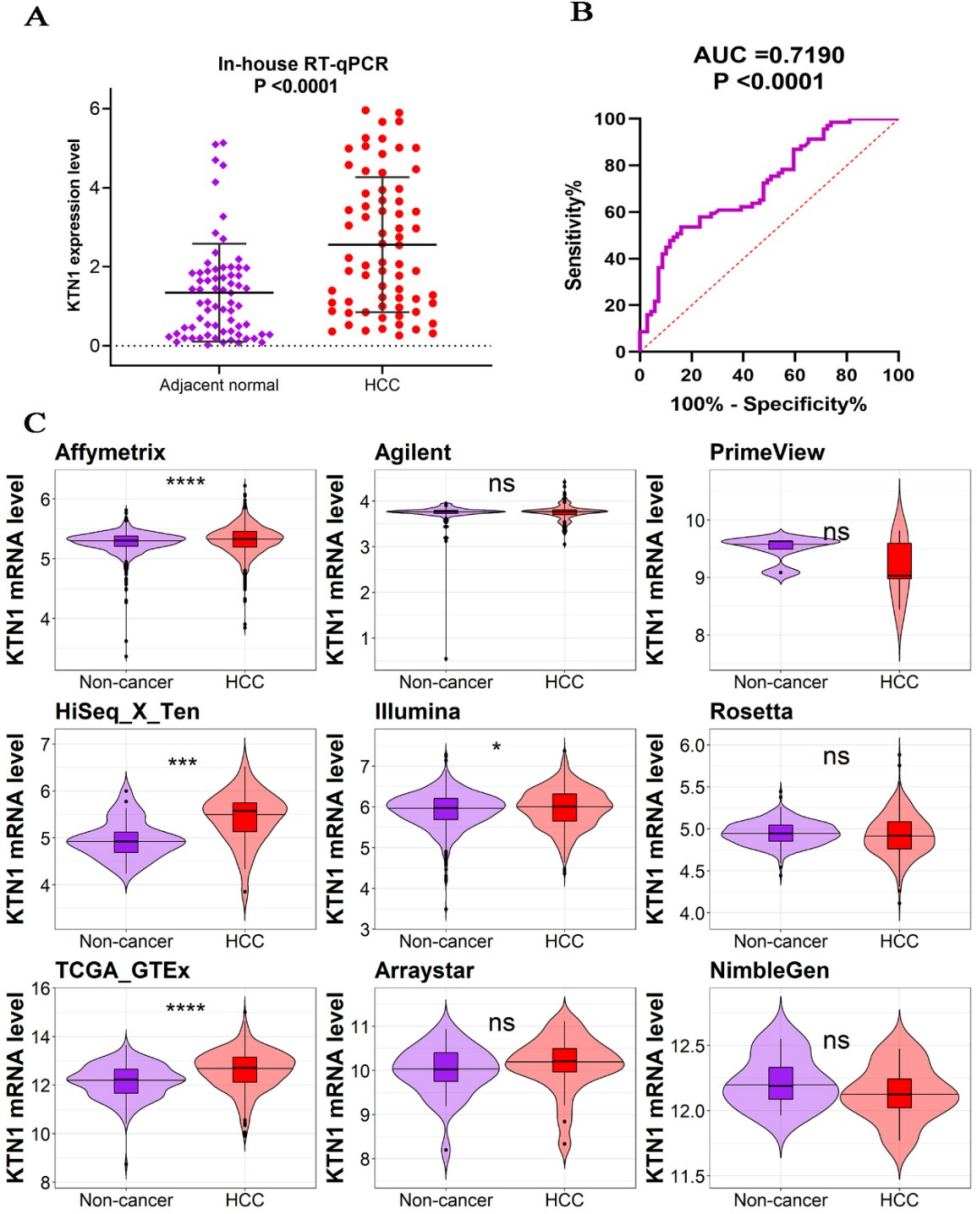
** Increased mRNA expression level of *KTN1* in HCC tissues. (A)** Result of the in-house reverse transcription real-time quantitative polymerase chain reaction (RT-qPCR) indicated *KTN1* expression was significantly increased in HCC. **(B)** A moderate discriminatory ability of *KTN1* between HCC and adjacent normal liver was shown by in-house RT-qPCR. **(C)** Validation of *KTN1* upregulation based on external datasets. *P <0.05, *** P <0.001, **** P <0.0001. HCC, hepatocellular carcinoma.

To clarify the expression status of* KTN1* and its prognostic value, this study quantitatively detected the expression level of *KTN1* and comprehensively analyzed the integrated gene chips and RNA-seq datasets. The results of in-house RT-qPCR indicated that *KTN1* was significantly upregulated in 50 HCC tissues compared to 50 pericarcinous tissues (Figure 1A) and that *KTN1* had a moderate ability to discriminate between HCC and non-HCC tissues (Figure 1B). External platform matrices integrated from gene chips and RNA-seq datasets covering 3,772 HCC and 2,998 non-HCC samples validated the upregulation trend of *KTN1* in HCC tissues (Table 1; Figure 1C) and the general discriminatory ability of *KTN1* (Figure S1). A pooled SMD value of 0.20 [0.04, 0.37] confirmed the increased expression level of *KTN1* in HCC tissues using a random-effect model (Figure S2A). The SMD value did not change dramatically when each platform was omitted (Figure S2B), and no publication bias influenced the results because P > 0.05 (Figure S2C). A moderate ability of *KTN1* to differentiate HCC from non-HCC tissue was detected from the SROC curve (Figure S3A), with a sensitivity (Figure S3B), specificity (Figure S3C), positive likelihood ratio (Figure S3D), and negative likelihood ratio (Figure S3E) of 44% [0.26, 0.63], 81% [0.67, 0.90], 2.36 [1.78, 3.12], and 0.69 [0.55, 0.86], respectively, implying its general accuracy.

### Potential pro-tumor role of *KTN1* in HCC

**Figure 2:**
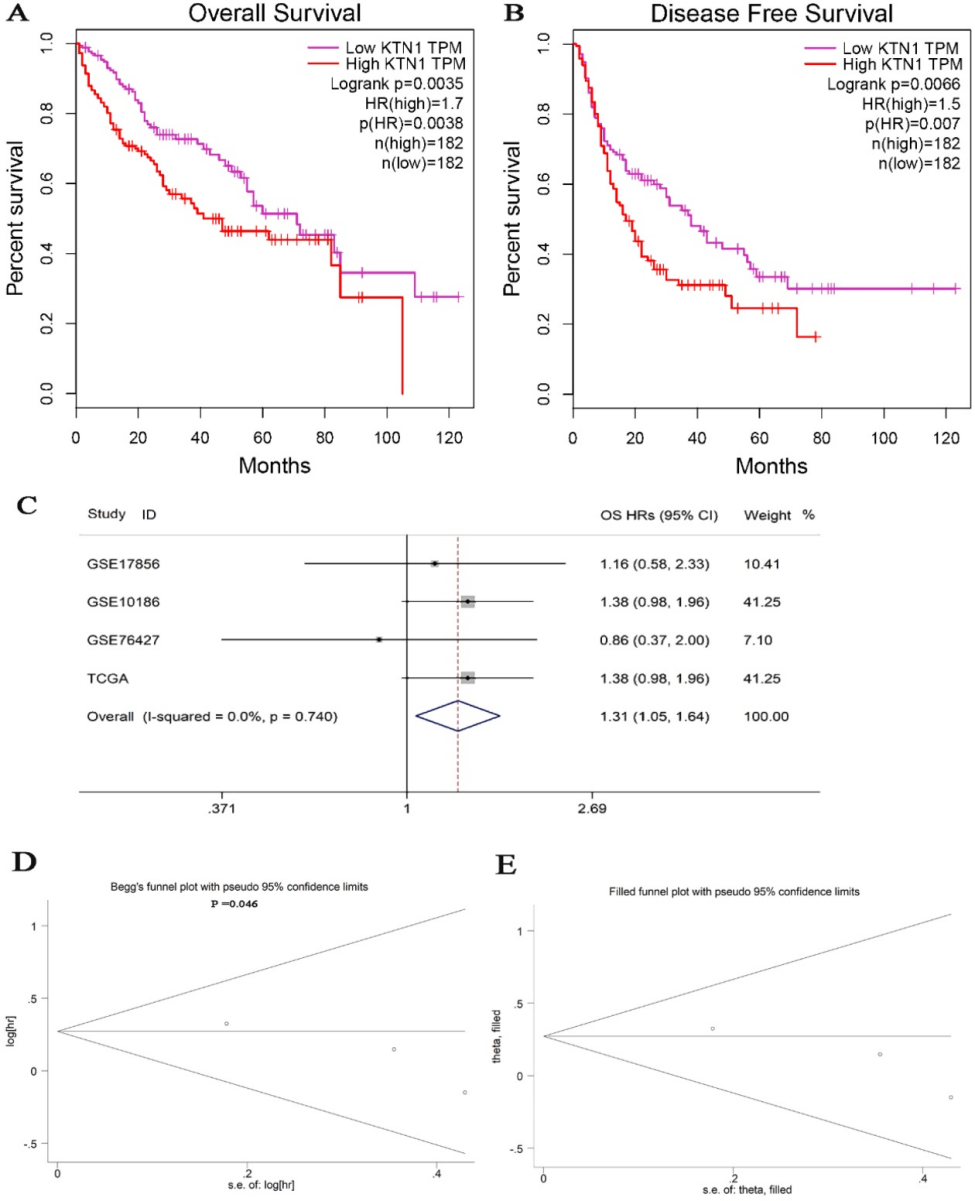
** Poor prognosis of *KTN1* expression in HCC. (A-B)** High *KTN1* expression predicted poor prognosis of overall survival and disease-free survival conditions of HCC patients. **(C)** Forest plot of overall survival hazard ratios signified *KTN1* was an independent risk factor of HCC patients. **(D)** Begg's funnel plot indicated a probability of publication bias. Therefore, Begg's funnel plot with trim and fill was also analyzed **(E)**. HCC, hepatocellular carcinoma.

**Figure 3:**
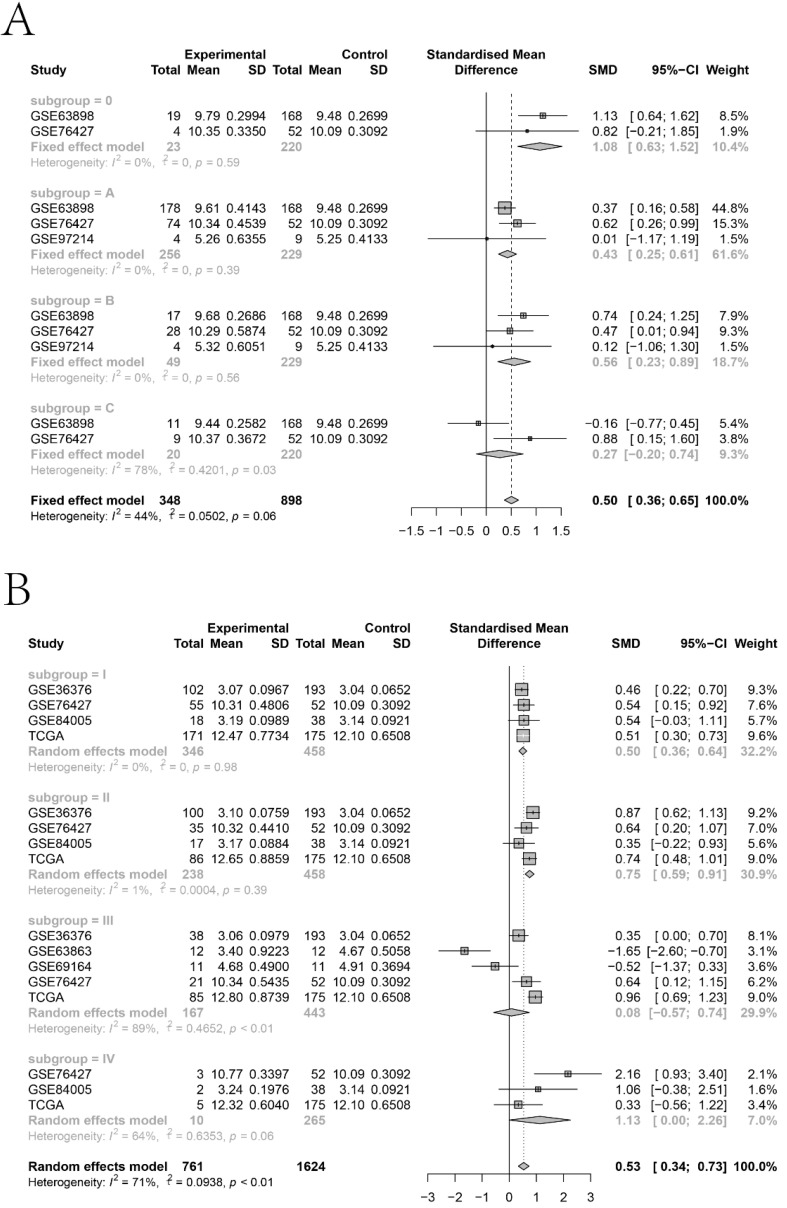
** Subgroup analysis based on the included hepatocellular carcinoma patients. (A)** Barcelona Clinic Liver Cancer staging subgroup. **(B)** TNM staging subgroup.

The *KTN1* high-*KTN1*-expression group in HCC had poorer overall survival and disease-free survival conditions than the low-*KTN1*-expression group (Figure 2A-B). A combined HR value of 1.31 [1.05, 1.64] indicated that *KTN1* may be an independent risk factor for HCC (Figure 2C). Trimmed and filled Begg's funnel plots showed the stability of the pooled HR results (Figure 2D-E). Table S1 presents the clinical features of the enrolled HCC patients. Higher *KTN1* was correlated with worse Child-Pugh classification grades and lower plasma α-fetoprotein (AFP) levels (Figure S4A-B). Therefore, *KTN1* might be an indicator in concealed HCC patients with low AFP expression. Additionally, the expression levels of *KTN1* varied from patient to patient. Specifically, *KTN1* was significantly upregulated in HCC patients at the zero, A, and B stages in terms of the BCLC system (Figure 3A-B). Interestingly, the upregulation of *KTN1* tended to be more obvious in stage-IV HCC patients, with a single SMD of 1.13. However, since the sample size of stage-IV HCC patients was limited, no significance was detected. Metastatic HCC gene chips were analyzed after removing the inter-study batch effect (Figure S5A-B). *KTN1* upregulation was a general phenomenon in both localized HCC tissues and metastatic HCC tissues (Figure S5C).

### Decreased expression of *KTN1* after knockout in Huh7 cell lines

**Figure 4:**
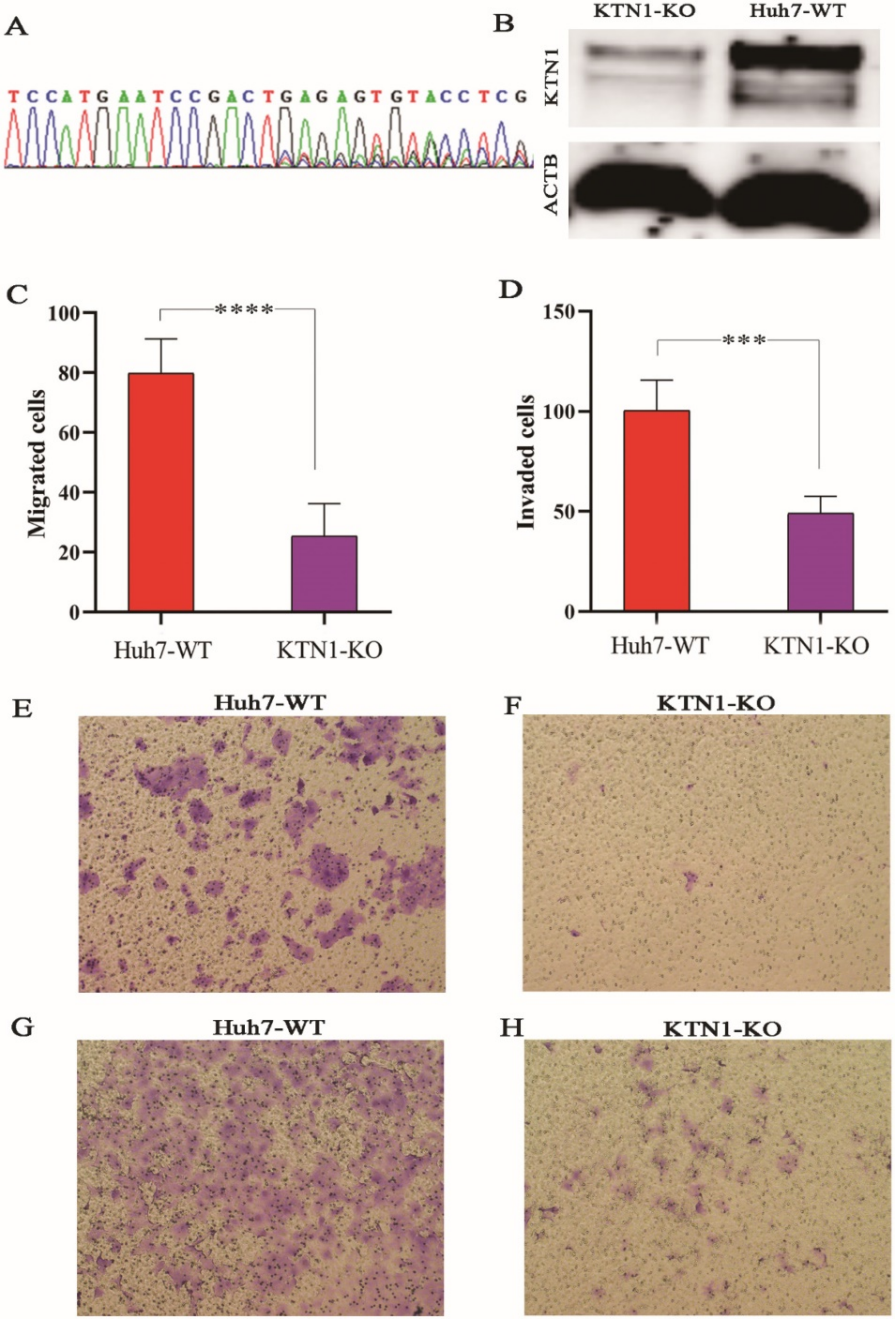
** CRISPR/Cas9-mediated *KTN1* knockout and biological functions of *KTN1* in HCC cells. (A)** Result of PCR-sanger sequencing verified the knockout efficiency. An obvious set of peaks was observed at the gRNA binding site, indicating the occurrence of a frame shift mutation. **(B)** Western blotting result verified the knockout efficiency. *KTN1* protein expression was decreased in *KTN1*-knock-out cells and ACTB was used as internal reference. **Panels C, E, and F** showed that the migration ability was attenuated in *KTN1*-knock-out cells as opposed to Huh7 cells. **Panels D, G, and H** showed that the invasion ability was attenuated in *KTN1*-knock-out cells as opposed to Huh7 cells. Therefore, *KTN1* could promote the migration and invasion of HCC cells. HCC, hepatocellular carcinoma.

To explore the biological functions of *KTN1* in HCC, the human cell line Huh7 was used to conduct a CRISPR/Cas9-mediated KTN1 knockout. The results of PCR-Sanger sequencing showed an obvious set of peaks at the gRNA binding site, indicating the occurrence of a frame-shift mutation, which confirmed the knockout efficiency (Figure 4A). Western blotting was performed, and decreased KTN1 protein expression was detected, further verifying the knockout efficiency (Figure 4B).

### Cell migration and invasion assays

As observed in Figure 4C-H, the migration and invasion abilities were attenuated in *KTN1*-KO cells compared to Huh7-WT cells, suggesting that *KTN1* promotes the migration and invasion of HCC cells.

### Cell-viability assay

**Figure 5:**
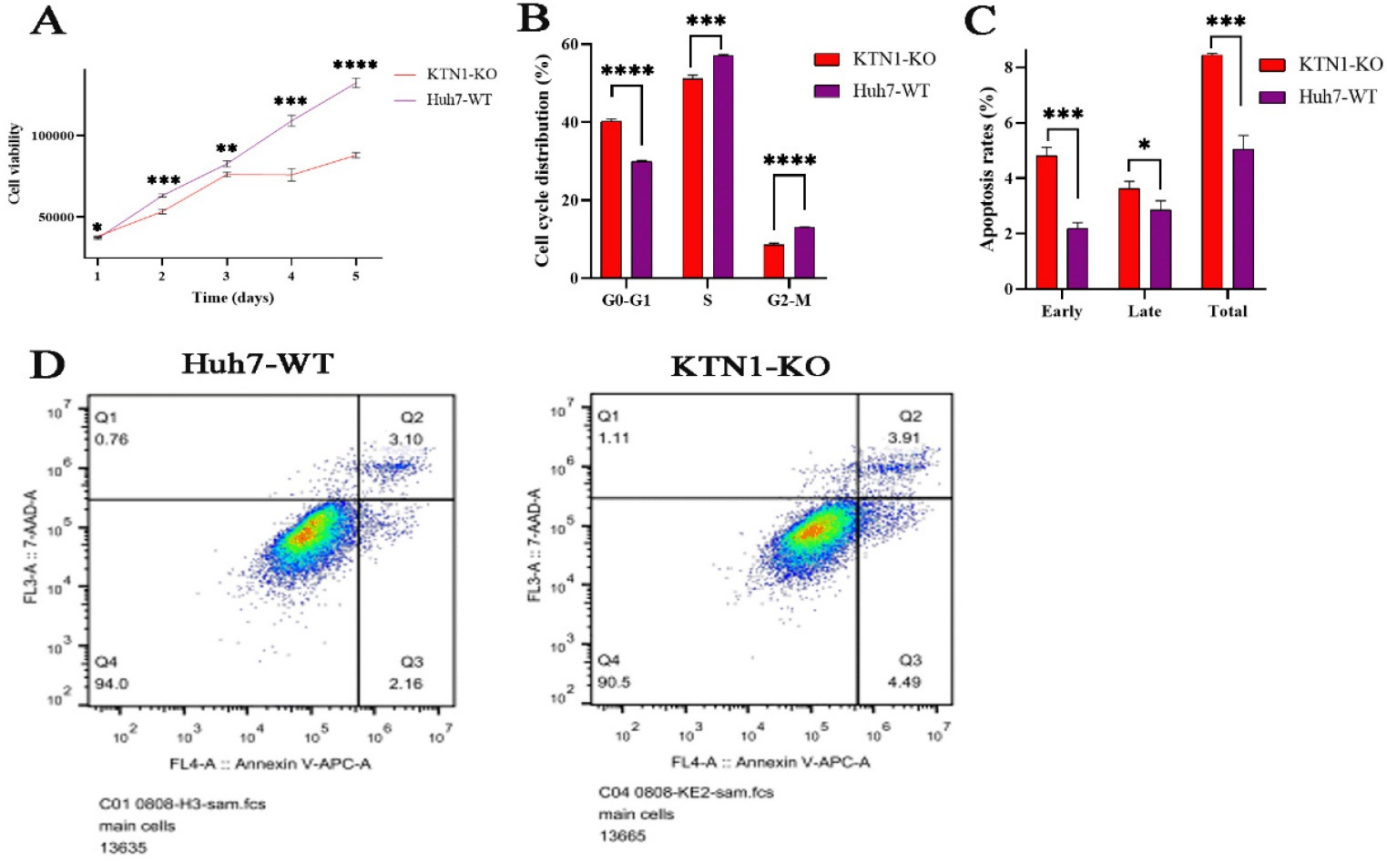
** Biological functions of *KTN1* in HCC. (A)** HCC cell viability was inhibited after *KTN1* knockout. **(B)** The proportion of HCC cells in G0-G1 phase increased after *KTN1* knockout, suggesting that *KTN1* knockout would block the transition from G1 to S phase. **(C)** *KTN1* knockout significantly increased the apoptosis rates in HCC cells in comparison with Huh7 cells, indicating *KTN1* could attenuate apoptosis of HCC cells. **Panel (D)** presented the early and late apoptotic cell ratios. HCC, hepatocellular carcinoma.

To determine the impact of *KTN1* on cell viability, this study continuously detected the relative viability of *KTN1*-KO cells and Huh7-WT cells within five days of knocking out *KTN1*. The results signified that cell viability was inhibited in *KTN1*-KO cells compared to Huh7-WT cells (Figure 5A), indicating that *KTN1* could enhance the viability of HCC cells.

### Cell cycle and apoptosis assays

The relationships between *KTN1*, cell cycle regulation, and cell apoptosis were investigated. The proportion of HCC cells in the G0-G1 phases increased after *KTN1* knockout, suggesting KTN1that it blocked the transition from the G1-S phases (Figure 5B). Moreover, *KTN1* knockout significantly increased the apoptosis rates in HCC cells in comparison to Huh7-WT cells, indicating that *KTN1* could attenuate the apoptosis of HCC cells (Figure 5C). Figure 5D presents higher early and late apoptotic cell ratios in *KTN1*-KO cells compared to Huh7-WT cells. Therefore, *KTN1* may accelerate cell cycle progression and hinder apoptosis in HCC cells.

### RNA-seq results

#### Quantitative expression analysis

**Figure 6:**
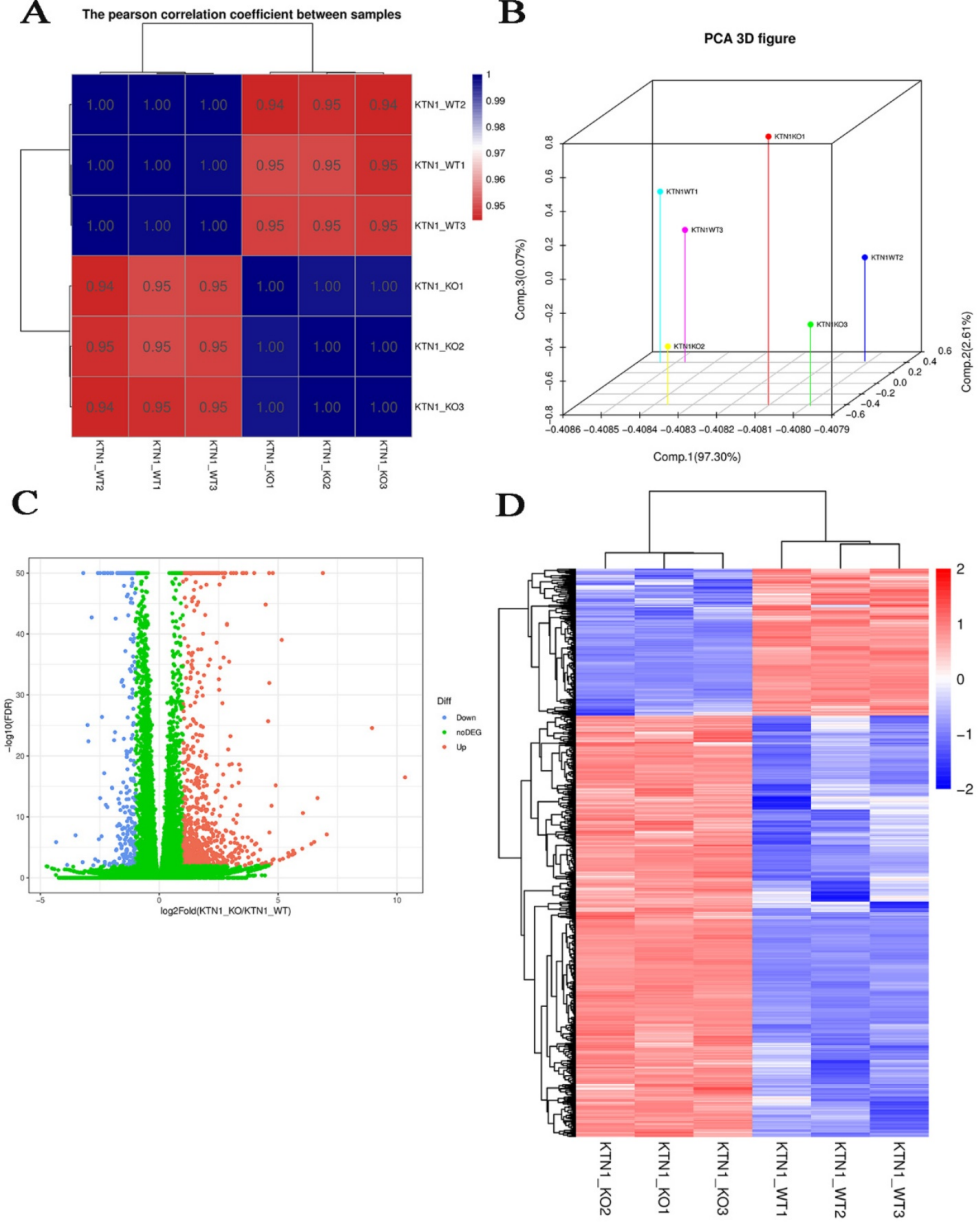
** Quantitative gene expression analysis by RNA-seq. (A)** Correlation analysis between samples. **(B)** Principal component analysis results. Each point in the figure represents a sample **(C)** DEGs between *KTN*1-knockout and Huh7-wildtype samples. The X-axis represents the fold change of DEGs, and the Y-axis represents the significance. Red represents upregulated genes, and blue represents downregulated genes. **(D)** DEGs heat map. DEGs, differently expressed genes.

Since *KTN1* plays an important role in multiple cellular functions of HCC, this study sought to determine the molecular events of *KTN1* underlying HCC. RNA-seq was conducted to analyze gene expressions and structures. Figure S6 displays the processes of transcriptome resequencing experiments and transcriptome resequencing analysis. High-correlation coefficients between samples indicated little variation among *KTN1*-KO samples, confirming the reproducibility of the experiment (Figure 6A). The results of principal component analysis signified that *KTN1* knockout was a reason for different expression levels (Figure 6B). In total, 793 upregulated genes and 277 downregulated genes were identified from the volcano plot (Figure 6C). A cluster analysis was used to compare the DEGs between different samples (Figure 6D).

#### GO, WEGO, and pathway-enrichment analysis

**Figure 7:**
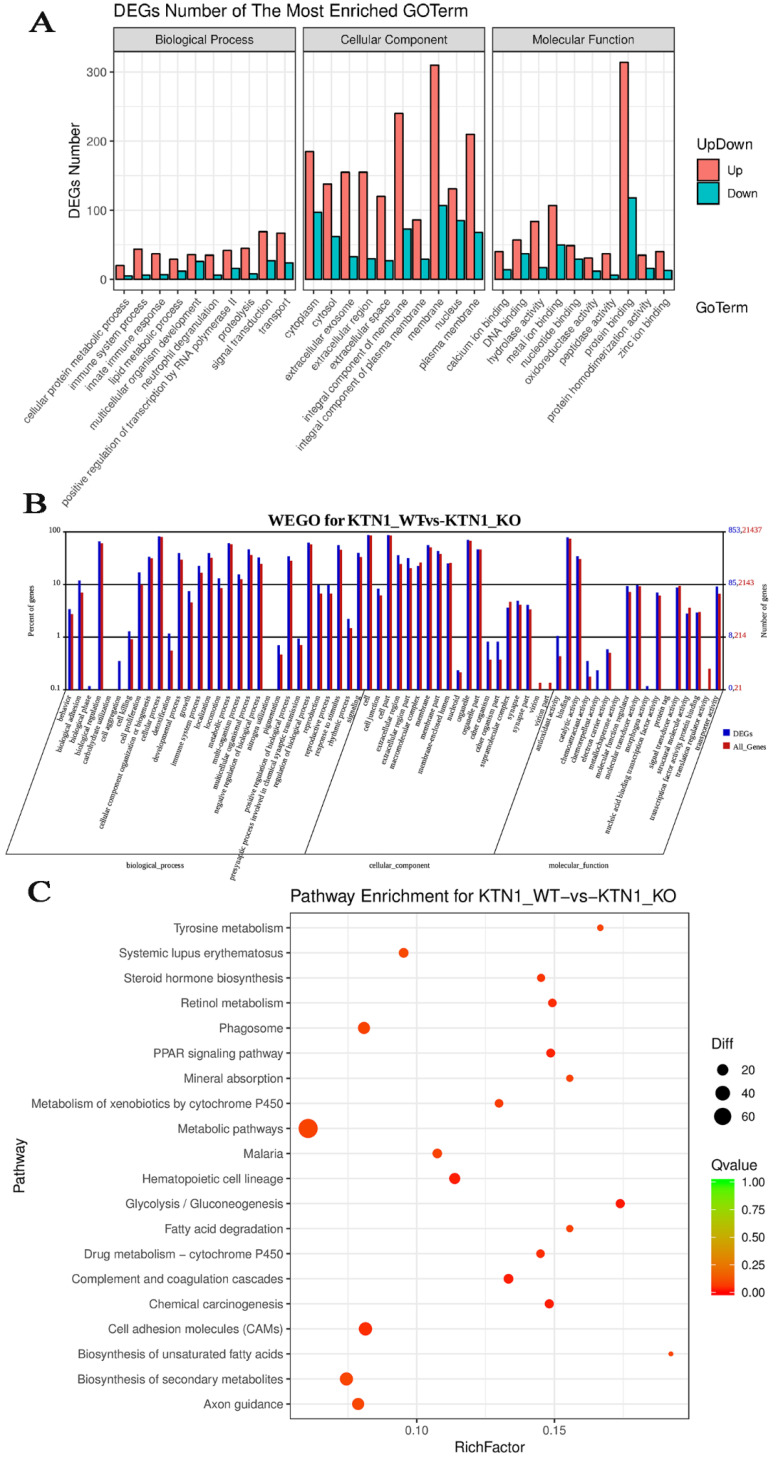
** Functional annotation based on the DEGs between *KTN*1-knockout and Huh7-wildtype samples. (A)** Gene ontology enrichment analysis. **(B)** WEGO analysis. The left Y-axis represents the ratio of gene numbers in each GO term and that in all GO terms. The left Y-axis represents the number of genes corresponding to GO term, where the red number represents the background (all genes) and the blue number represents the differential gene. **(C)** Kyoto Encyclopedia of Genes and Genomes pathway. DEGs, differently expressed genes.

To explore the potential mechanisms of *KTN1*, functional annotation was based on the DEGs between *KTN1*-KO cells and Huh7-WT cells, which after *KTN1* knockout, were predominantly enriched in signal transduction, transport, immune system process, innate immune response, and positive regulation of transcription by RNA polymerase II in GO biological processes (Figure 7A). Interestingly, “integral component of membrane” was one of the most aggregated GO cellular component terms, which corresponded to the renowned nature of *KTN1* as an integral membrane protein. In addition, DNA binding, protein binding, and metal ion binding (i.e., Ca^2+^ and Zn^2+^) were the dominant GO molecular function terms. After being normalized by the background, WEGO analysis showed that translation regulator activity, virion, biological phase, cell aggregation, chemorepellent activity, and morphogen activity were the most altered GO terms after the *KTN1* knockout *KTN1* (Figure 7B). Furthermore, chemical carcinogenesis, metabolic pathways (e.g., tyrosine metabolism, metabolism of xenobiotics by cytochrome P50, and drug metabolism), and cell-adhesion molecules were the most clustered pathways (Figure 7C).

#### Identification of risk-prediction signatures

**Figure 8:**
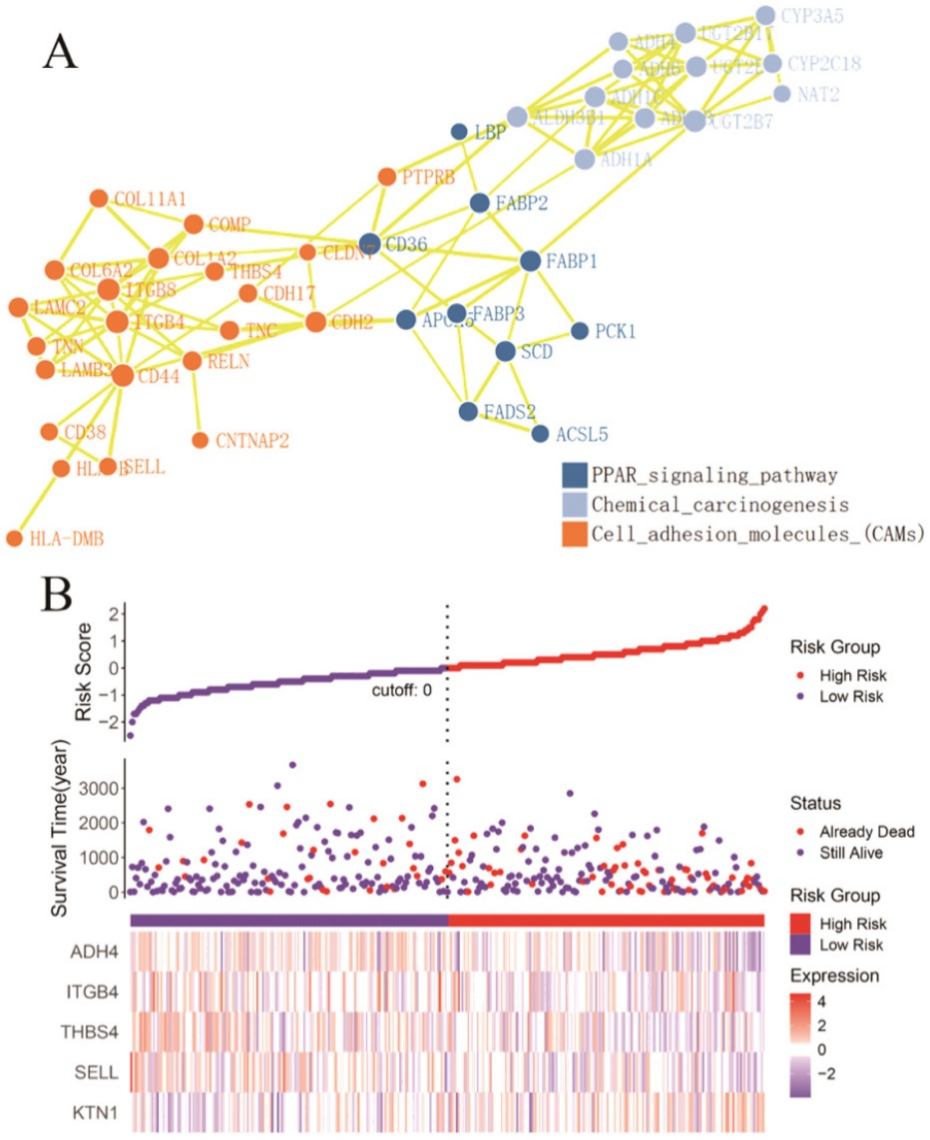
** Identification of *KTN1* associated risk prediction signatures in HCC. (A)** Protein-protein interaction network based on the chemical carcinogenesis, PPAR signaling pathway, retinol metabolism, and cell adhesion molecules (Note: genes that enriched in the retinol metabolism pathway all participated in the chemical carcinogenesis pathways). **(B)** Risk prediction of HCC patients based on the *KTN1* associated risk prediction signatures. HCC, hepatocellular carcinoma.

**Figure 9:**
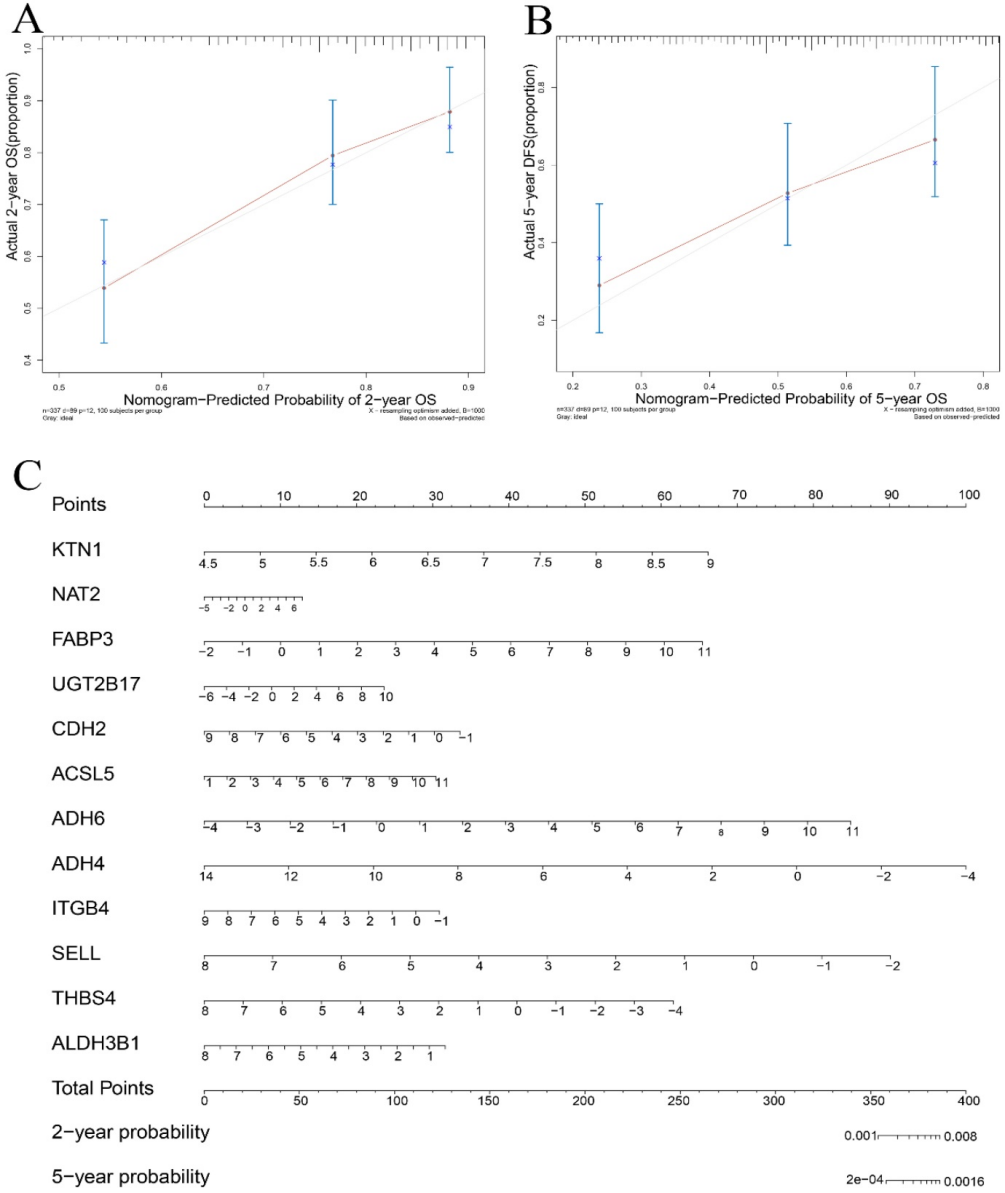
** Validation of the risk prediction model in HCC. (A-B)** Calibration curve based on the *KTN1* associated risk prediction signatures in HCC. **(C)** Nomogram in predicting the 2-year and 5-year survival condition of HCC patients. HCC, hepatocellular carcinoma.

A total of 54 genes aggregated in the chemical carcinogenesis, PPAR signaling pathway, retinol metabolism, and cell-adhesion molecules (Figure 8A) were used to perform a Cox proportional-hazard regression analysis based on the TCGA cohort. Figure 8B presents the risk distribution of the HCC patients. *SELL*, *ITGB4*, *ALDH3B1*, *THBS4*, and *ADH4* were predicted to be protective factors of HCC, while *UGT2B17*,* NAT2*,* FABP3*,* CDH2*,* ACSL5*, and* ADH6* were forecasted as risk factors of HCC (Table S2). Interestingly, *ITGB4* and *UGT2B17* were hub genes of the cell-adhesion molecules and retinol metabolism pathways. Combined with *KTN1*, these risk-predicting signatures showed moderately accurate indications of the 2-year and 5-year survival conditions of HCC patients, with a C-index of 0.7010 [se(C) = 0.0317] (Figure 9A-C). However, more studies are needed to validate the accuracy of the risk model.

### Co-expression regulatory network analysis

**Figure 10:**
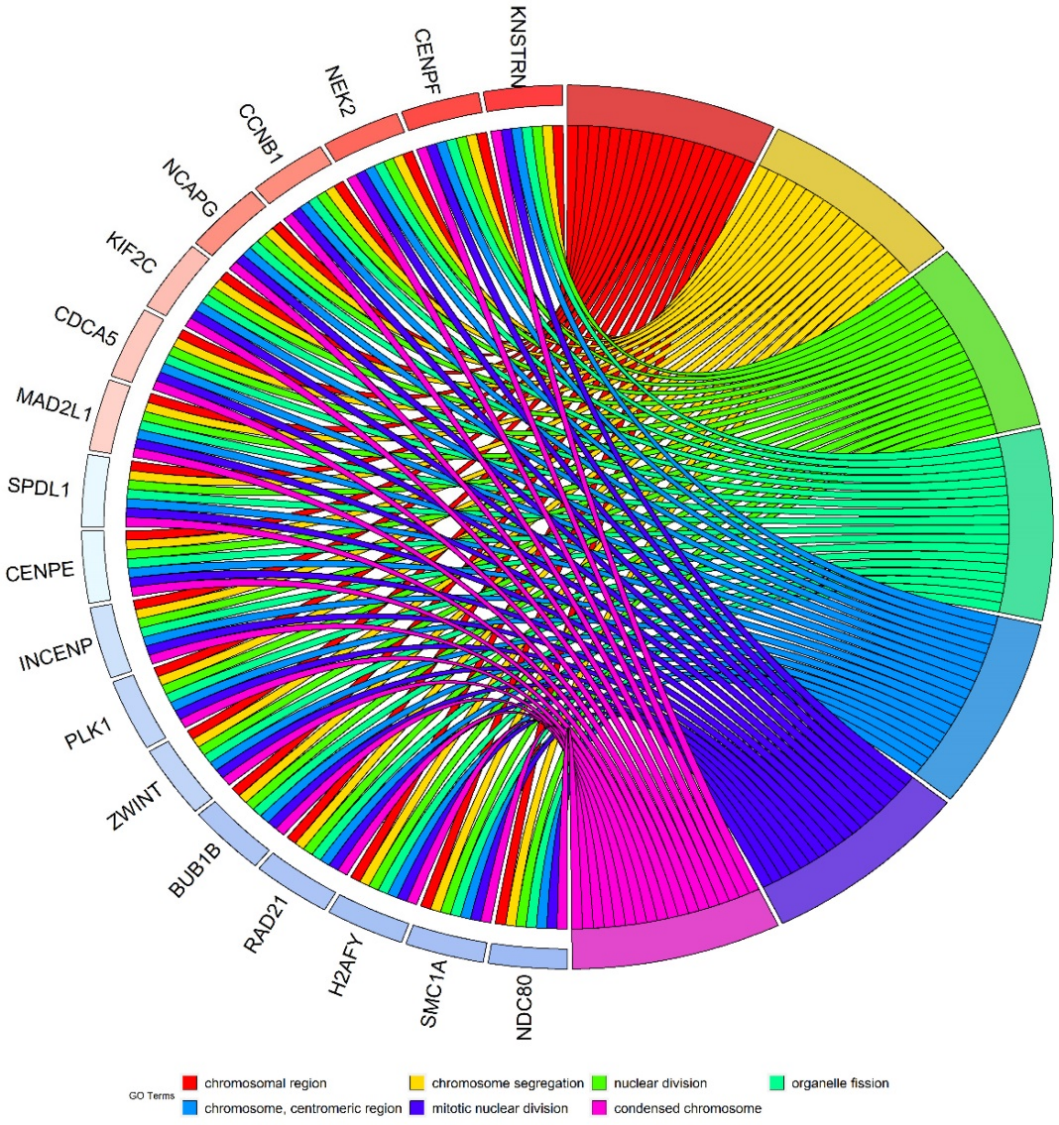
** Gene ontology enrichment analysis based on the intersected genes of DGEs in HCC and CEGs positively related to *KTN1*.** DEGs, differentially expressed genes; CEGs, co-expressed genes; HCC, hepatocellular carcinoma.

**Figure 11:**
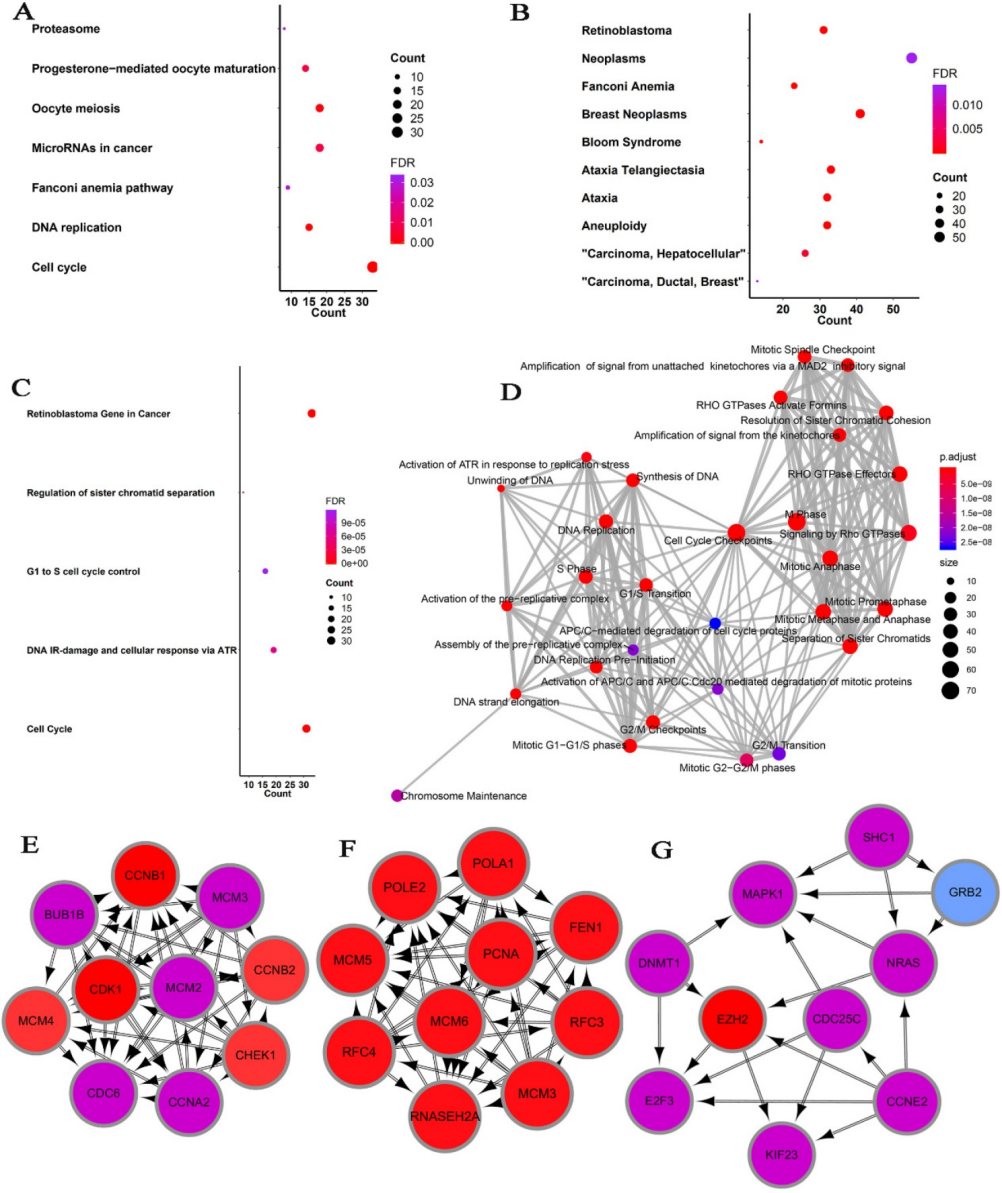
** Pathway analysis based on the intersected genes of DEGs in HCC and CEGs that related to* KTN1*. (A)** Kyoto Encyclopedia of Genes and Genomes pathway. **(B)** Disease Ontology. **(C)** Wikipathway. **(D)** Reactome pathway. **(E-F)** Protein-to-protein interaction networks based on the KEGG pathways of cell cycle, DNA replication, and microRNAs in cancer. DEGs, differentially expressed genes; HCC, hepatocellular carcinoma; CEGs, co-expressed genes.

Considering the possibility of distinct mechanisms of *KTN1* in HCC cell lines and human tissues, the potential functions of *KTN1* in HCC tissues were annotated. In total, 1,499 upregulated genes and 6,872 CEGs positively related to *KTN1* were identified from the integrated platform expression matrices. Figure S7A-B shows two expression heatmaps based on the Arraystar and NimbleGen platforms. By intersecting upregulated genes and CEGs positively related to *KTN1*, 687 overlapping genes were obtained that were significantly enriched in chromosome segregation, nuclear division, organelle fission, microtubule binding, chromatin binding, and tubulin binding in terms of GO (P < 0.05, Figure 10; Table S3). In addition, the cell cycle, DNA replication, oocyte meiosis, and microRNAs in cancer were the top four most aggregated KEGG pathways (P < 0.05, Figure 11A; Table S4). Pertaining to disease annotation, the significantly enriched diseases were HCC, retinoblastoma, and breast neoplasms (P < 0.05, Figure 11B; Table S5). Moreover, the retinoblastoma gene in cancer, cell cycle, regulation of sister chromatid separation, and the G1-S cell cycle control were the significant WikiPathways (P < 0.05, Figure 11C; Table S6). Furthermore, the upregulated genes related to *KTN1* in HCC were pronouncedly accumulated in cell cycle checkpoints, the separation of sister chromatids, and DNA replication in terms of Reactome pathways (P < 0.05, Figure 11D; Table S7). PPI networks were constructed to display the co-expression relationships among genes in the cell cycle, DNA replication, and microRNAs of cancer pathways, respectively (Figure 11E-G). *CCNB1*, *MCM6*,* EZH2*,* CDK1*, *MCM3*, *POLE2*, *POLA1*, *MCM5*, *RNASEH2A*,* FEN1*, *RFC4*, *PCNA*, and* RFC3* were identified as core genes, and nine of them (i.e., *CCNB1*, *MCM6*, *EZH2*, *CDK1*, *MCM3*, *POLA1*, *RNASEH2A*, *FEN1*, and *RFC4*) were risk factors in HCC patients (all with HRs > 1, P < 0.05).

## Discussion

HCC is one of the most heterogeneous and pernicious tumors in the world 34-36. The knowledge gap between *KTN1* and HCC necessitates more research to clarify the roles of *KTN1* and to direct future development of novel therapies for HCC. In this study, *KTN1* was comprehensively analyzed from multi-faceted perspectives for the first time, covering clinical tissues, *in vitro* experiments, and *in silico* analysis. This study was prospective on several levels. First, the increased expression levels of *KTN1* in HCC were determined by integrated analysis*KTN1* and confirmed by in-house RT-qPCR and large external datasets containing gene chips and RNA-seq data with a total of 6,870 participants. In addition, this study enrolled HCC patients from all over the world after careful screening, which makes the results more convincing. Second, the clinical implications of *KTN1* were fully evaluated by a retrospective cohort study. High *KTN1* expression was significantly correlated to poorer prognosis of HCC patients, and *KTN1* may be an independent risk factor for HCC. Third, the biological functions of *KTN1* were confirmed by high-quality *in vitro* experiments using the CRISPR/Cas9 gene-editing system. Excitingly, *KTN1* may promote migration and invasion ability, enhance cell viability, accelerate cell cycle progression, and hinder apoptosis in HCC cells. Importantly, a risk-prediction model for HCC patients was established based on the DEGs identified from the in-house RNA-seq. Fourth, the complicated molecular mechanisms of *KTN1* underlying HCC were revealed on the cell and tissue levels by analyzing in-house RNA-seq results combined with *in silico* annotations. Several cascades including innate immune response, chemical carcinogenesis, and positive regulation of transcription by RNA polymerase II, were dramatically changed after *KTN1* knockout. Moreover, in human HCC tissues, *KTN1* may participate in the cell cycle, DNA replication, and microRNAs in cancer pathways. Therefore, this study has pinpointed a novel role of *KTN1* in HCC development.

*KTN1* was upregulated in HCC tissues and correlated with unfavorable outcomes in HCC patients. This study provided convincing evidence of the increased expression of *KTN1* by analyzing a total of 3,822 HCC tissues and 3,048 non-HCC tissues, results that have not been reported before. Given the upregulated expression pattern of *KTN1*, the researchers speculate that it has applications in HCC screening and prognosis surveillance. Although the discriminatory ability of *KTN1* between HCC and non-HCC tissues was general, the prognostic value of *KTN1* in HCC deserves further verification in clinical practice in the future. The increased expression of *KTN1* forecasted the poorer survival conditions of HCC patients, and *KTN1* may be an independent risk factor for HCC. Therefore, the potential clinical value of *KTN1* in HCC is promising, but more studies are required to promote its applications.

*KTN1* was successfully knocked out in a Huh7 cell line using the CRISPR/Cas9 gene-editing system, revealing the biological functions of *KTN1* in HCC cells. *In vitro* experiments, including a cell-viability assay, a migration assay, a cell invasion assay, a cell-cycle assay, and an apoptosis assay, determined the impact of *KTN1* on HCC at the cell level. Surprisingly, the migration and invasion abilities and cell viability were attenuated in *KTN1*-KO cells compared to Huh7-WT cells, indicating that *KTN1* may enhance cell viability and promote cell migration and invasion in HCC. A previous study reported the role of *KTN1* in contributing to the direction of cell migration in breast cancer cells 22, and another disturbed the interaction between KTN1 proteins and kinesin and observed that cell migration was hindered 37. Such studies point to the promotion effect of *KTN1* on cells. Furthermore, *KTN1* knockout raised the proportion of HCC cells in the G0-G1 phases and increased apoptosis rates, suggesting that *KTN1* promotes cell-cycle progression but inhibits cell apoptosis in HCC. These findings support *KTN1*'s important role in the malignant manifestations of HCC by regulating cell viability, migration, invasion, cell cycle activation, and apoptotic inhibition. In-depth *in vivo* experiments must be performed to confirm this result.

The genetic expression changes in HCC cells after *KTN1* knockout were quantitatively detected by RNA-seq. In this study, a total of 793 upregulated genes and 277 downregulated genes were identified in the *KTN1*-KO cells, which were aggregated in immune activities (i.e., immune system process and innate immune response), gene expression (i.e., positive regulation of transcription by RNA polymerase II and translation regulator activity), and chemical carcinogenesis. Interestingly, the KTN1 protein, as mentioned in the Introduction, influenced transcription by binding to EF-δ and participating in the assembly of the EF-1 complex, which was consistent with the annotation result in this study. Furthermore, *KTN1* was confirmed to be a pathogenic autoimmune antigen in several diseases 38, 39, suggesting its role *KTN1*in regulating immune systems and being involved in tumor immunity. More importantly, the authors revealed an association between *KTN1* and the chemical carcinogenesis pathway, which may be responsible for the onset of HCC. Overall, the *KTN1* knockout experiment and RNA-seq analysis make it reasonable to propose that *KTN1* induces the development of HCC cells by transcription regulation, immune mechanisms, and chemical carcinogenesis.

The potential molecular mechanisms of *KTN1* underlying HCC tissues were functionally annotated. Surprisingly, upregulated genes that co-expressed with *KTN1* in HCC tissues were significantly enriched in cell cycle and cell-division-related biological processes (i.e., chromosome segregation, nuclear division, organelle fission, microtubule binding, chromatin binding, and tubulin binding). Specifically, the authors observed that such genes were significantly clustered in the G1-S cell cycle control pathway, which was consistent with the *in vivo* experimental evidence mentioned in Section 3.5. To the best of our knowledge, KTN1 proteins and kinesin are microtubule-associated proteins that participate in cell division 40-43. In tumor cells, KTN1 proteins and kinesin may be abused. Therefore, *KTN1* indeed plays an essential role in promoting cell cycle G1/S transition and may induce uncontrollable the cell division and malignant biological behaviors of HCC, resulting in its progression. Moreover, such genes were correlated to HCC in terms of disease enrichment, further supporting our proposal.

Furthermore, *KTN1* may be a candidate target for developing novel therapies for HCC patients, one reason for which is that *KTN1* had immune activity. As mentioned in the Introduction, *KTN1* was the most frequently detected antigen in a serological analysis based on HCC tissue samples 23. More interestingly, even molecules with tandem-repeat features like the KTN1 protein (i.e., CDR34 and CT7) also reacted with the serum antibodies from tumor patients 44, 45. Nonetheless, more research is required to mine the therapeutic value of *KTN1* in HCC.

This study faced several limitations, of course. First, it did not probe the potential sources of high heterogeneity among different platforms. To minimize the interruption effect of the heterogeneity-to-SMD result, a random-effect model was utilized. Second, the biological effect and pro-tumor role of *KTN1* in HCC was not verified on the *in vivo* level. However, to reveal the functions of *KTN1* in HCC, this study performed *in vitro* experiments on *KTN1*-KO cells using the CRISPR/Cas9 gene-editing system, biological assays, and RNA-seq data. Our study also provided new directions for research on the mechanism of HCC progression, although more experimental evidence must be found to verify the pro-tumor role of *KTN1*. Third, *in silico* functional annotation was conducted to explore possible molecular mechanisms, for which further *in vivo* studies are required to support our findings.

## Conclusions

*KTN1* was upregulated in HCC and served as a promising prognosticator in patients. *KTN1* may induce the occurrence and deterioration of HCC by mediating cell survival, migration, invasion, cell cycle activation, and apoptotic inhibition.

## Supplementary Material

Supplementary figures and tables.

## Figures and Tables

**Table 1:** Statistical information of the external gene chip and RNA sequencing datasets

Platform	HCC			Non-HCC			P	TP	FP	FN	TN
	N1	M1	SD1	N2	M2	SD2					
Affymetrix	1656	5.3148	0.2330	1356	5.2789	0.1702	<0.0001*	485	157	1171	1199
Agilent	186	3.7549	0.1676	131	3.7241	0.3041	0.2493	19	0	167	131
PrimeView	5	9.1724	0.5436	5	9.4958	0.2363	0.2573	3	0	2	5
HiSeq X Ten	35	5.4274	0.5524	35	4.9672	0.4064	0.0002*	27	9	8	26
Illumina	1117	5.9772	0.4806	822	5.9258	0.4293	0.0133*	258	102	859	720
Rosetta	368	4.9271	0.2448	386	4.9482	0.1366	0.1476	109	39	259	347
TCGA-GTEx	371	12.6055	0.8301	225	12.1540	0.6789	<0.0001*	246	82	125	143
Arraystar	26	10.1334	0.6367	30	9.9886	0.5679	0.3723	21	17	5	13
NimbleGen	8	12.1336	0.2197	8	12.2219	0.2027	0.4177	5	3	3	5

*P <0.05 indicated significance.

## Abbreviations

HCC: hepatocellular carcinoma
*KTN1*: kinectin 1
EF: elongation factor
CSCC: cutaneous squamous cell carcinoma
RT-qPCR: reverse transcription real-time quantitative-polymerase chain reaction
RNA-seq: RNA sequencing
SMD: standardized mean difference
SROC: summary receiver operating characteristic
HRs: hazard ratios
WT: wild-type
KO: knockout
FPKM: fragment Per Kilo bases per Million fragment
DEGs: differentially expressed genes
GO: Gene Ontology
KEGG: Kyoto Encyclopedia of Genes and Genomes
CEGs: co-expressed genes
DO: Disease Ontology
PPI: protein-protein-interaction
