# Supplementary material for: Upregulating KTN1 promotes Hepatocellular Carcinoma progression
Source: J Cancer. 2021 Jun 11;12(16):4791–809. doi: 10.7150/jca.55570 (PMC8247380; doi:10.7150/jca.55570)
Supplement: Supplementary file 1 — Supplementary figures and tables. [file jcav12p4791s1.pdf]

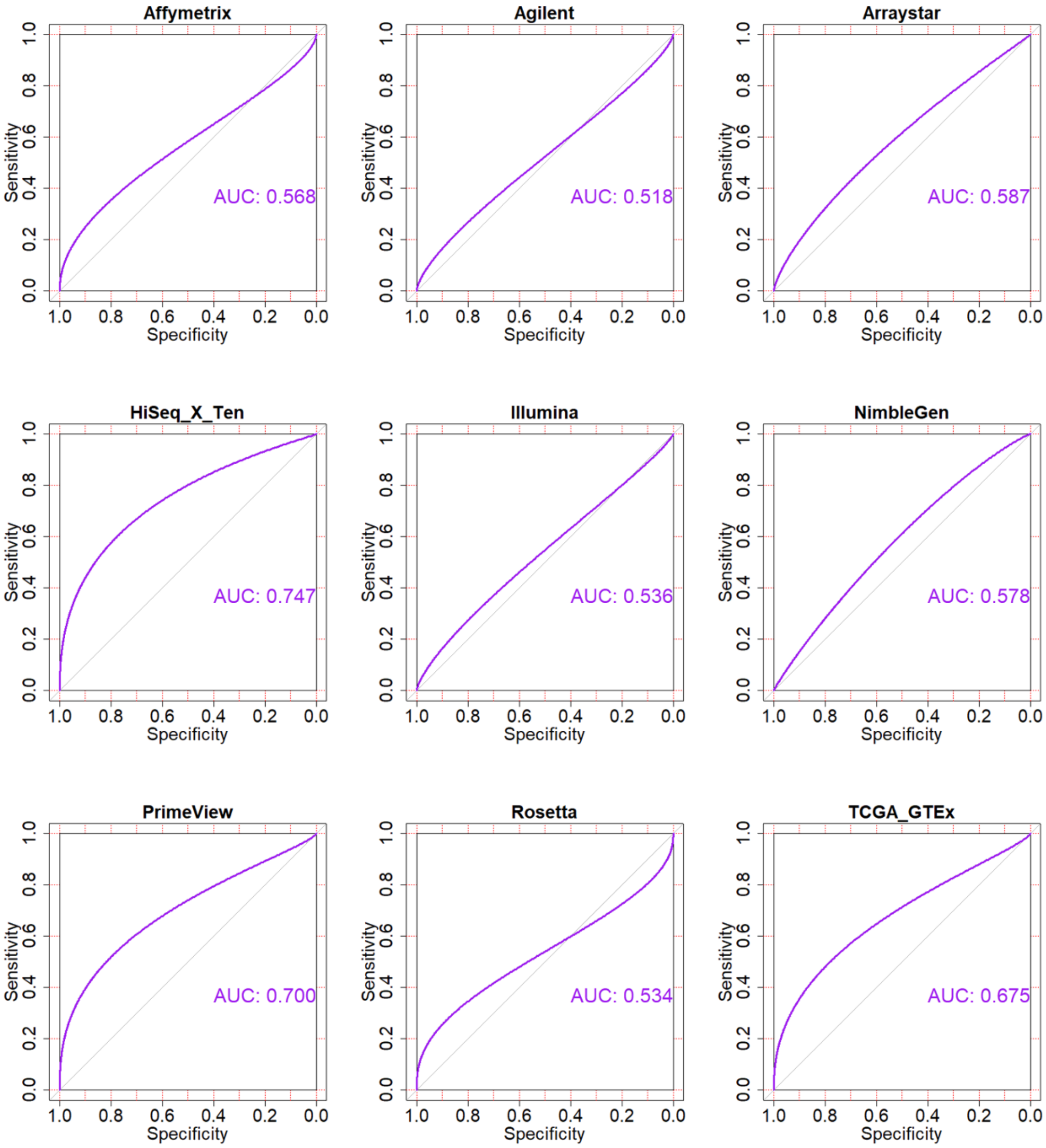
Affymetrix
AUC: 0.568
Agilent
AUC: 0.518
Arraystar
AUC: 0.587
HiSeq_X_Ten
AUC: 0.747
Illumina
AUC: 0.536
NimbleGen
AUC: 0.578
PrimeView
AUC: 0.700
Rosetta
AUC: 0.534
TCGA_GTEx
AUC: 0.675
Sensitivity
Specificity
1.0
0.8
0.6
0.4
0.2
0.0

Figure S1

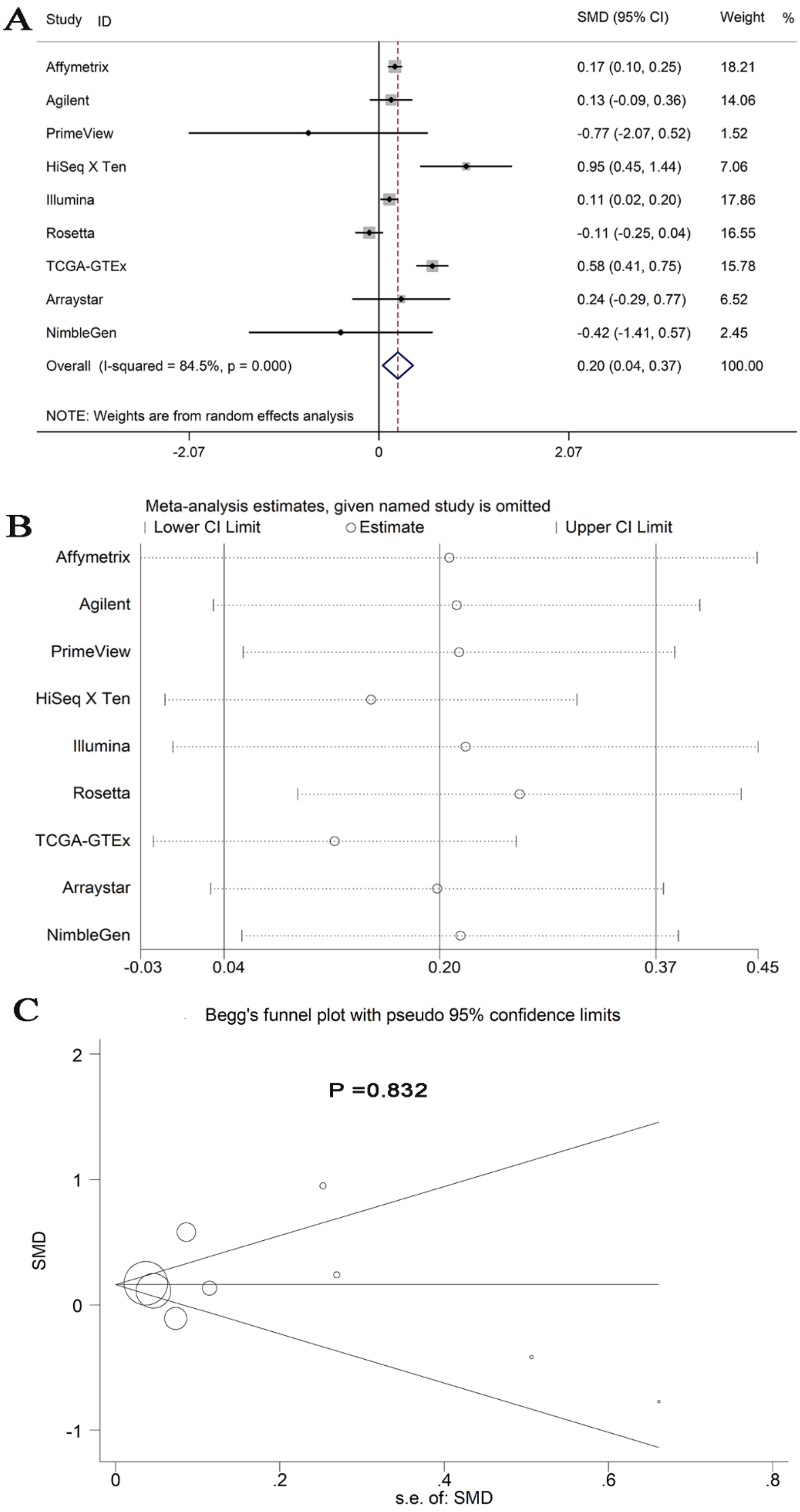
A
Study ID
SMD (95% CI)
Weight %
Affymetrix
0.17 (0.10, 0.25)
18.21
Agilent
0.13 (-0.09, 0.36)
14.06
PrimeView
-0.77 (-2.07, 0.52)
1.52
HiSeq X Ten
0.95 (0.45, 1.44)
7.06
Illumina
0.11 (0.02, 0.20)
17.86
Rosetta
-0.11 (-0.25, 0.04)
16.55
TCGA-GTEx
0.58 (0.41, 0.75)
15.78
Arraystar
0.24 (-0.29, 0.77)
6.52
NimbleGen
-0.42 (-1.41, 0.57)
2.45
Overall (I-squared = 84.5%, p = 0.000)
0.20 (0.04, 0.37)
100.00
NOTE: Weights are from random effects analysis
-2.07
0
2.07
B
Meta-analysis estimates, given named study is omitted
Lower CI Limit
Estimate
Upper CI Limit
Affymetrix
Agilent
PrimeView
HiSeq X Ten
Illumina
Rosetta
TCGA-GTEx
Arraystar
NimbleGen
-0.03
0.04
0.20
0.37
0.45
C
Begg's funnel plot with pseudo 95% confidence limits
P =0.832
SMD
2
1
0
-1
0
.2
.4
.6
.8
s.e. of: SMD

Figure S2

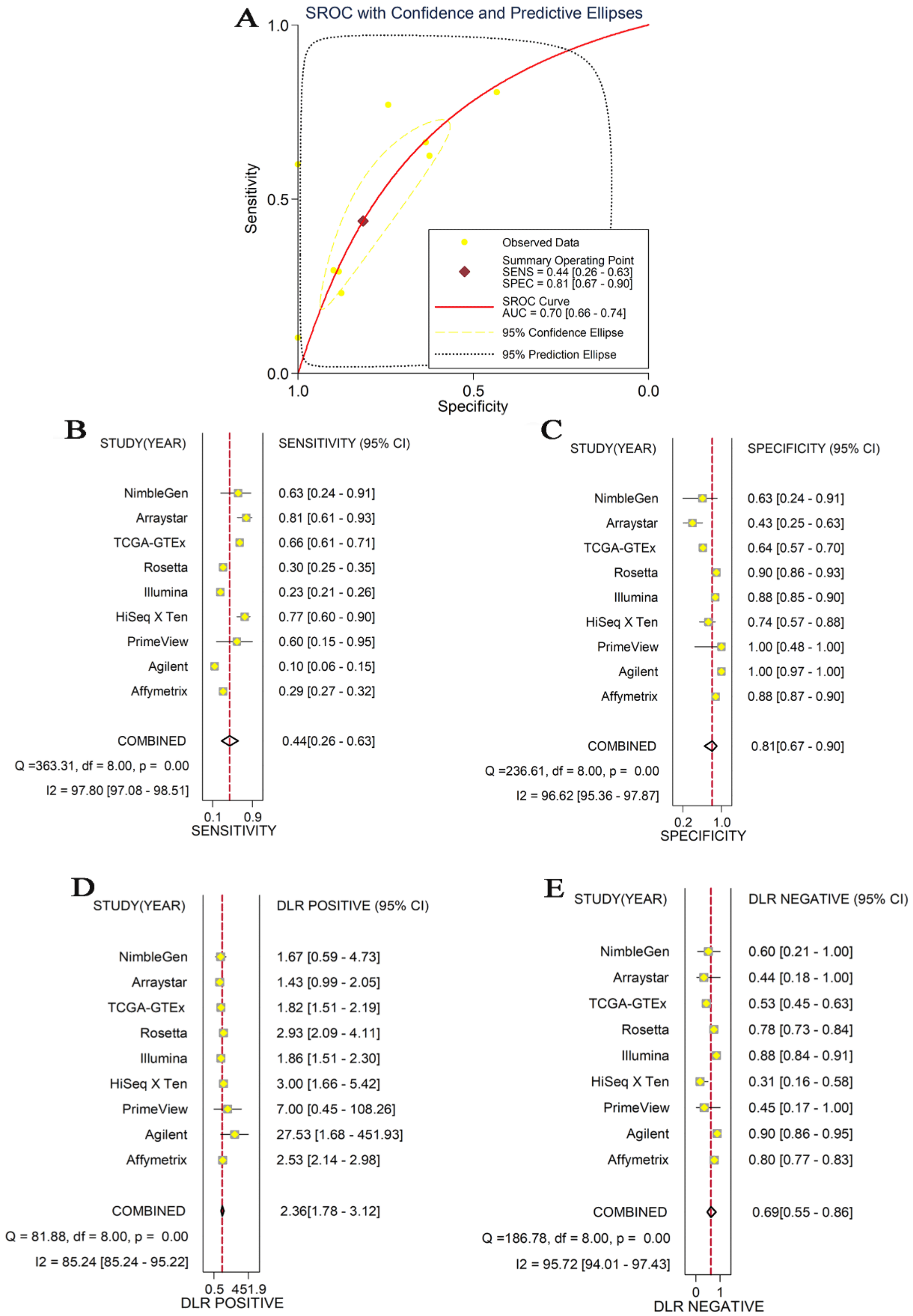

A
SROC with Confidence and Predictive Ellipses
Sensitivity
Specificity
Observed Data
Summary Operating Point
SENS = 0.44 [0.26 - 0.63]
SPEC = 0.81 [0.67 - 0.90]
SROC Curve
AUC = 0.70 [0.66 - 0.74]
95% Confidence Ellipse
95% Prediction Ellipse
B
STUDY(YEAR)
SENSITIVITY (95% CI)
NimbleGen 0.63 [0.24 - 0.91]
Arraystar 0.81 [0.61 - 0.93]
TCGA-GTEx 0.66 [0.61 - 0.71]
Rosetta 0.30 [0.25 - 0.35]
Illumina 0.23 [0.21 - 0.26]
HiSeq X Ten 0.77 [0.60 - 0.90]
PrimeView 0.60 [0.15 - 0.95]
Agilent 0.10 [0.06 - 0.15]
Affymetrix 0.29 [0.27 - 0.32]
COMBINED 0.44[0.26 - 0.63]
Q =363.31, df = 8.00, p = 0.00
I2 = 97.80 [97.08 - 98.51]
SENSITIVITY
C
STUDY(YEAR)
SPECIFICITY (95% CI)
NimbleGen 0.63 [0.24 - 0.91]
Arraystar 0.43 [0.25 - 0.63]
TCGA-GTEx 0.64 [0.57 - 0.70]
Rosetta 0.90 [0.86 - 0.93]
Illumina 0.88 [0.85 - 0.90]
HiSeq X Ten 0.74 [0.57 - 0.88]
PrimeView 1.00 [0.48 - 1.00]
Agilent 1.00 [0.97 - 1.00]
Affymetrix 0.88 [0.87 - 0.90]
COMBINED 0.81[0.67 - 0.90]
Q =236.61, df = 8.00, p = 0.00
I2 = 96.62 [95.36 - 97.87]
SPECIFICITY
D
STUDY(YEAR)
DLR POSITIVE (95% CI)
NimbleGen 1.67 [0.59 - 4.73]
Arraystar 1.43 [0.99 - 2.05]
TCGA-GTEx 1.82 [1.51 - 2.19]
Rosetta 2.93 [2.09 - 4.11]
Illumina 1.86 [1.51 - 2.30]
HiSeq X Ten 3.00 [1.66 - 5.42]
PrimeView 7.00 [0.45 - 108.26]
Agilent 27.53 [1.68 - 451.93]
Affymetrix 2.53 [2.14 - 2.98]
COMBINED 2.36[1.78 - 3.12]
Q = 81.88, df = 8.00, p = 0.00
I2 = 85.24 [85.24 - 95.22]
DLR POSITIVE
E
STUDY(YEAR)
DLR NEGATIVE (95% CI)
NimbleGen 0.60 [0.21 - 1.00]
Arraystar 0.44 [0.18 - 1.00]
TCGA-GTEx 0.53 [0.45 - 0.63]
Rosetta 0.78 [0.73 - 0.84]
Illumina 0.88 [0.84 - 0.91]
HiSeq X Ten 0.31 [0.16 - 0.58]
PrimeView 0.45 [0.17 - 1.00]
Agilent 0.90 [0.86 - 0.95]
Affymetrix 0.80 [0.77 - 0.83]
COMBINED 0.69[0.55 - 0.86]
Q =186.78, df = 8.00, p = 0.00
I2 = 95.72 [94.01 - 97.43]
DLR NEGATIVE

Figure S3

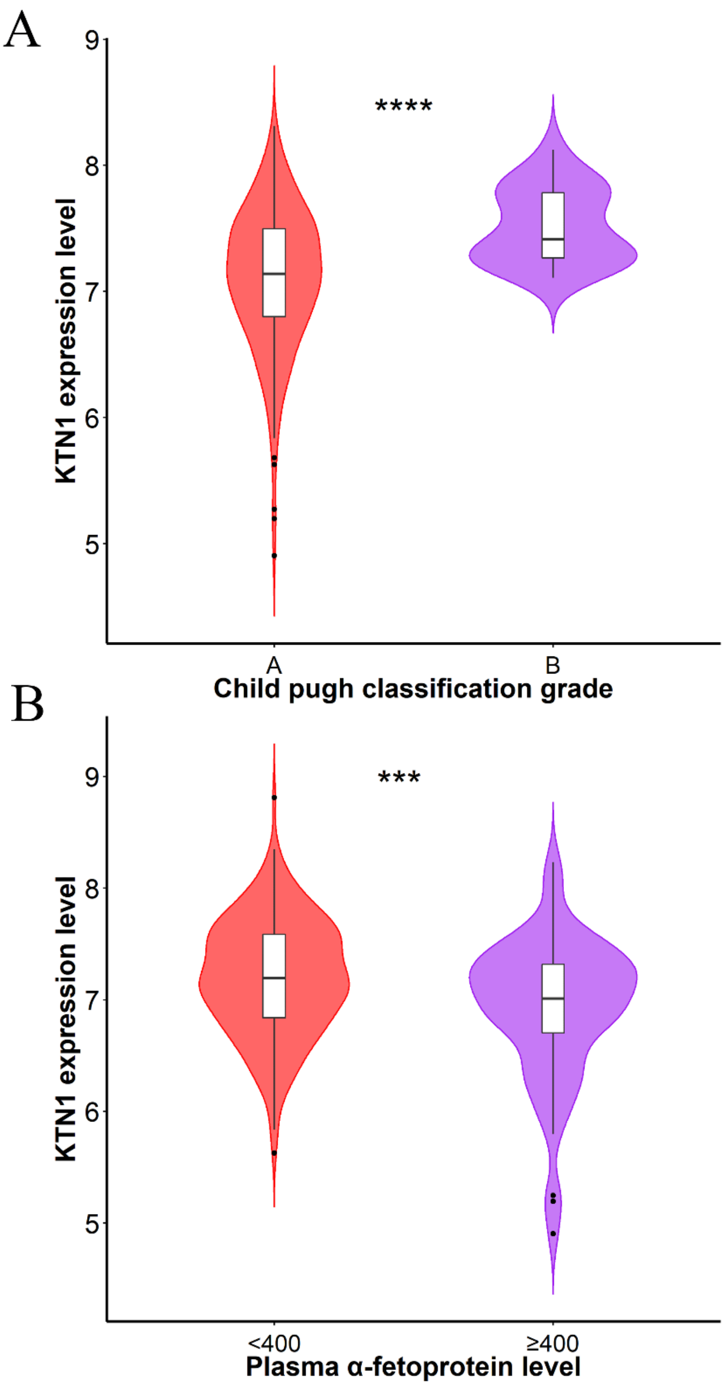
A
B
KTN1 expression level
****
***
Child pugh classification grade
Plasma α-fetoprotein level
<400
≥400
9
8
7
6
5

Figure S4

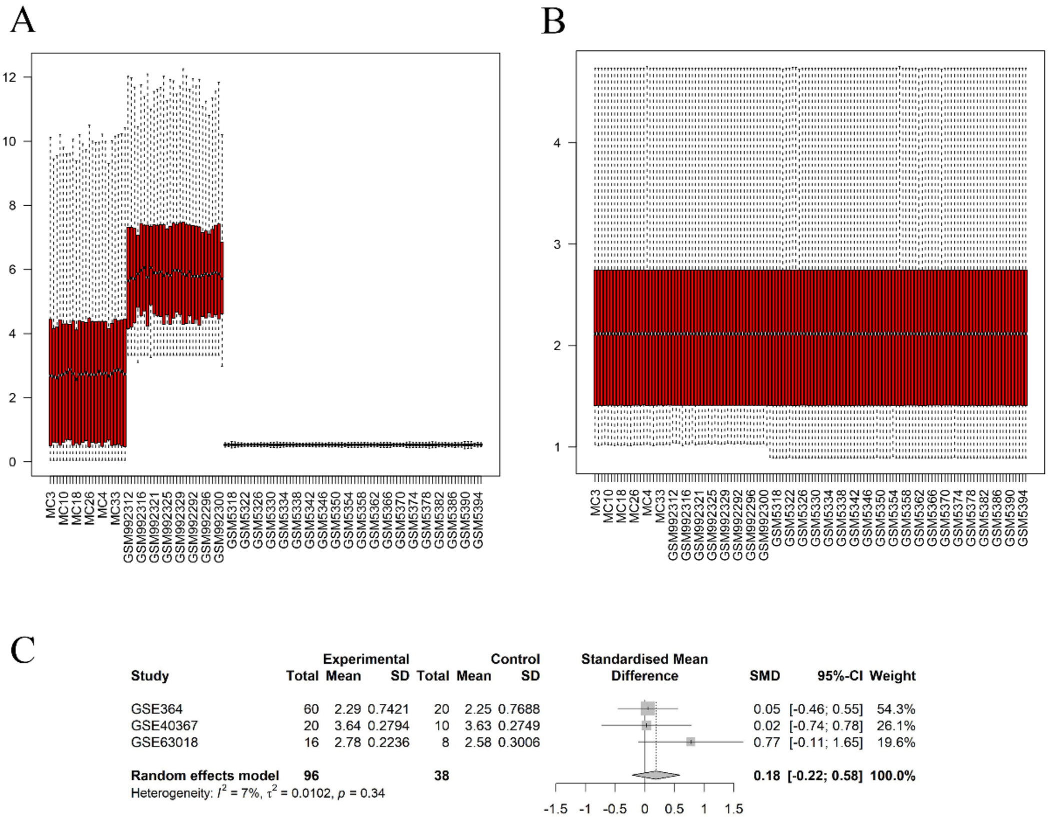
A
B
C
Study
Experimental Total Mean SD
Control Total Mean SD
Standardised Mean Difference
SMD 95%-CI Weight
GSE364 60 2.29 0.7421 20 2.25 0.7688 0.05 [-0.46; 0.55] 54.3%
GSE40367 20 3.64 0.2794 10 3.63 0.2749 0.02 [-0.74; 0.78] 26.1%
GSE63018 16 2.78 0.2236 8 2.58 0.3006 0.77 [-0.11; 1.65] 19.6%
Random effects model 96 38 0.18 [-0.22; 0.58] 100.0%
Heterogeneity: I² = 7%, τ² = 0.0102, p = 0.34
-1.5 -1 -0.5 0 0.5 1 1.5

Figure S5

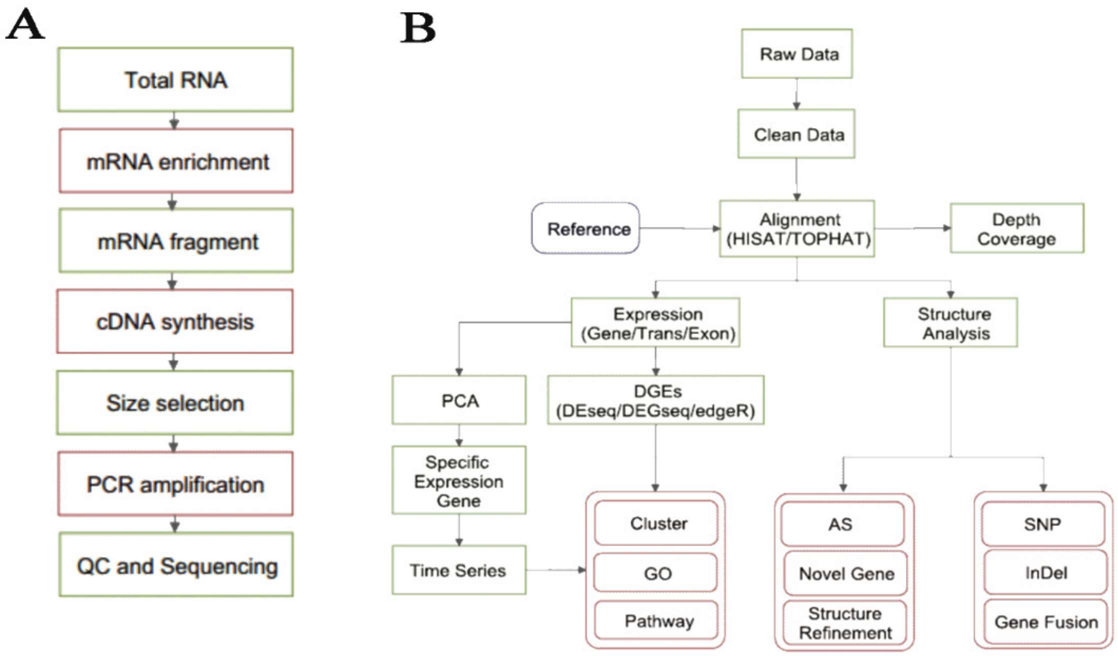
A
Total RNA
mRNA enrichment
mRNA fragment
cDNA synthesis
Size selection
PCR amplification
QC and Sequencing
B
Raw Data
Clean Data
Reference
Alignment (HISAT/TOPHAT)
Depth Coverage
Expression (Gene/Trans/Exon)
Structure Analysis
PCA
DGEs (DEseq/DEGseq/edgeR)
Specific Expression Gene
Time Series
Cluster
GO
Pathway
AS
Novel Gene
Structure Refinement
SNP
InDel
Gene Fusion

Figure S6

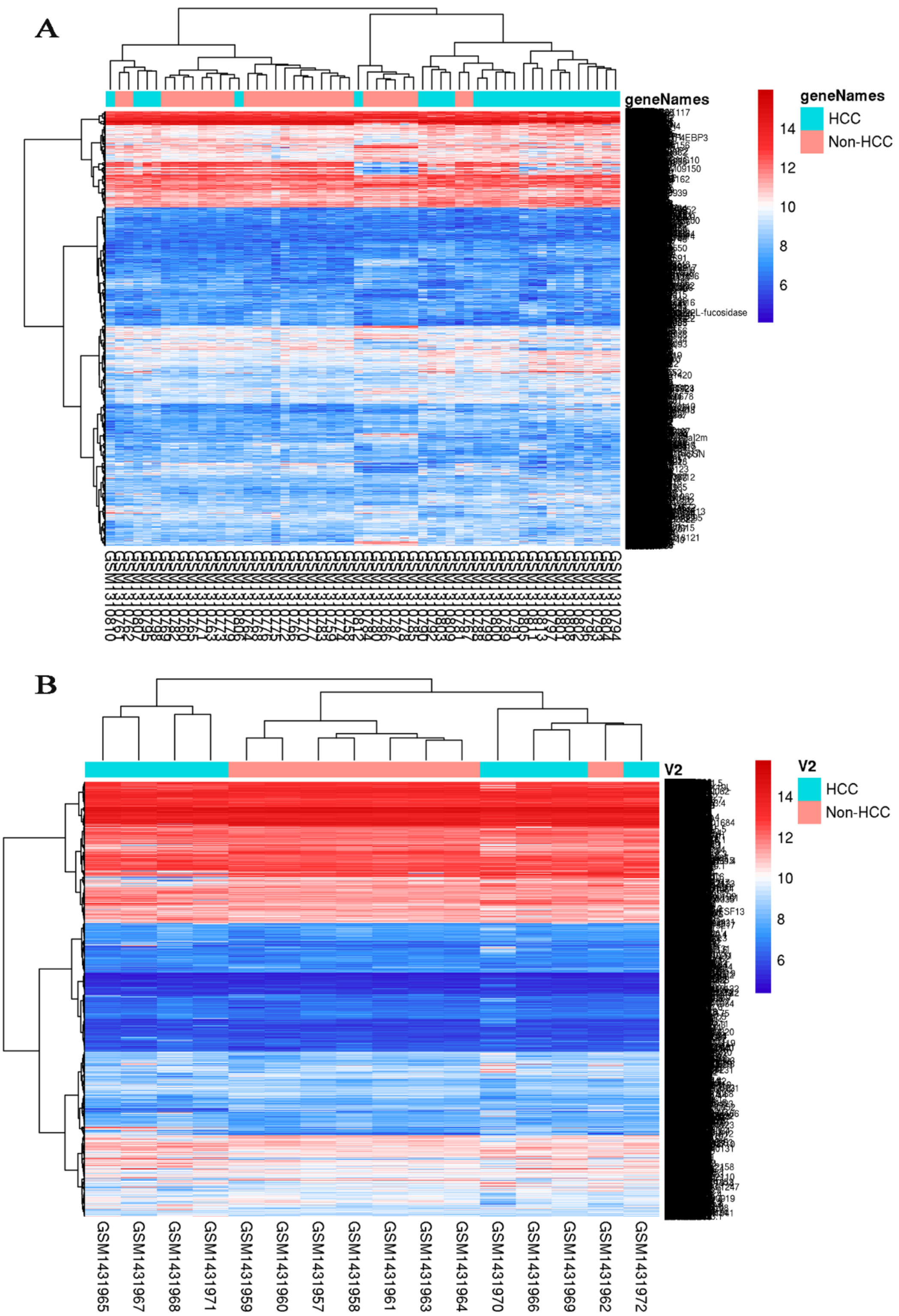
A
geneNames
HCC
Non-HCC
14
12
10
8
6
B
V2
GSM1431965
GSM1431967
GSM1431968
GSM1431971
GSM1431959
GSM1431960
GSM1431957
GSM1431958
GSM1431961
GSM1431963
GSM1431964
GSM1431970
GSM1431966
GSM1431969
GSM1431962
GSM1431972

Figure S7

Table S1: The clinic parameters of enrolled hepatocellular carcinoma patients.

Clinical parameters	Features	Relative expression level of *KTN1*				
		Total	%	M	SD	P
Gender	Female	121	32.61	7.13	0.65	0.53
	Male	249	67.12	7.17	0.53	
Age (years)	<60 (47.92±10.51)	169	45.68	7.14	0.53	0.66
	⩾60 (69.13±6.15)	201	54.32	7.17	0.61	
Height (cm)	166.84±9.65	/	/	/	/	/
Weight (kg)	68.30±18.53	/	/	/	/	/
Race	Non-Asian	206	56.59	7.10	1.04	
	Asian	158	43.41	7.10	0.56	0.99
Ethnicity	Not hispanic or latino	340	94.97	7.04	1.10	
	Hispanic or latino	18	5.03	7.26	0.51	0.41
Relative family cancer history	No	204	64.56	7.11	0.57	0.08
	Yes	112	35.44	7.23	0.59	
Cancer first degree relative	1	57	52.78	7.15	0.64	0.15
	⩾2	51	47.22	7.31	0.52	
History of HCC risk factors	No	91	25.85	7.15	0.65	0.79
	Yes	261	74.15	7.17	0.54	
Histologic grade	G1-G2	232	63.39	7.18	0.57	0.30
	G3-G4	134	36.61	7.11	0.58	
Histologic grade	G1	55	15.03	7.20	0.54	*
	G2	177	48.36	7.17	0.58	
	G3	122	33.33	7.16	0.58	
	G4	12	3.28	6.68	0.38	
Residual tumor	R0	324	95.01	7.14	0.58	0.60
	R1	17	4.99	7.22	0.68	
Vascular invasion	No	206	65.40	7.17	0.52	0.45
	Yes	109	34.60	7.11	0.66	
Child-Pugh classification grade	A	218	91.21	7.10	0.57	****
	B	21	8.79	7.52	0.30	
α-fetoprotein	<400	213	76.62	7.20	0.52	***
	⩾400	65	23.38	6.93	0.64	
Fibrosis	No	74	34.91	7.15	0.61	0.40
	Yes	138	65.09	7.22	0.55	
Fibrosis ishak score	No fibrosis	75	35.21	7.05	1.03	0.39
	Portal fibrosis	31	14.55	7.12	0.52	
	Fibrous speta	28	13.15	7.12	0.61	
	Nodular formation and incomplete cirrhosis	9	4.23	7.30	0.67	
	Established cirrhosis	70	32.86	7.29	0.52	
Adjacent hepatic tissue	No	117	50.00	7.14	0.66	0.45

inflammation	Yes	117	50.00	7.20	0.48	
Adjacent hepatic tissue	None	117	50.00	7.14	0.66	0.65
inflammation extent	Mild	99	42.31	7.18	0.47	
	Severe	18	7.69	7.27	0.51	
Pathologic stage	Stage I & II	257	74.06	7.15	0.57	0.48
	Stage III & IV	90	25.94	7.19	0.60	
Pathologic stage	Stage I	171	49.28	7.13	0.51	0.50
	Stage II	86	24.78	7.18	0.67	
	Stage III	85	24.50	7.21	0.60	
	Stage IV	5	1.44	6.89	0.51	
Tumor status	T1	181	49.18	7.13	0.50	0.61
	T2	94	25.54	7.21	0.67	
	T3	80	21.74	7.14	0.61	
	T4	13	3.53	7.29	0.57	
Lymph node status	N0	253	98.44	7.16	0.57	0.25
	N1	4	1.56	7.50	0.93	
Metastasis status	M0	265	98.51	7.15	0.61	0.52
	M1	4	1.49	6.95	0.57	

Table S2: Cox regression analysis based on *KTN1* associated differentially expressed genes.

**ID**	**Coefficient**	**exp(coef)**	**se(coef)**	**z**	**p**
SELL	-0.47345	0.622851	0.165322	-2.864	0.00419
ITGB4	-0.3989	0.671061	0.137063	-2.91	0.00361
ALDH3B1	-0.36785	0.692222	0.185096	-1.987	0.04688
THBS4	-0.23457	0.790911	0.082709	-2.836	0.00457
ADH4	-0.20489	0.814736	0.064717	-3.166	0.00155
UGT2B17	0.10629	1.112144	0.04796	2.216	0.02668
NAT2	0.178471	1.195388	0.072992	2.445	0.01448
FABP3	0.267172	1.306265	0.119012	2.245	0.02477
CDH2	0.288045	1.333817	0.138903	2.074	0.03811
ACSL5	0.349394	1.418207	0.169903	2.056	0.03974
ADH6	0.566341	1.761809	0.179008	3.164	0.00156

Table S3: Result of gene ontology enrichment analysis according to the upregulated genes co-expressed with *KTN1* in hepatocellular carcinoma.

**Term**	**ID**	**Description**	**P. adjusted**	**Gene**
BP	GO:0007059	chromosome segregation	<0.0001	ACTR3/TPR/RAD21/RAN/SMC1A/SRPK1/FANCD2/MKI67/CDCA5/BIRC5/H2AFY/CDC20/PLK1/DMC1/KIF2C/INCENP/CENPW/CDC6/FEN1/KIFC1/HJURP/CDCA8/BUB1B/NCAPH/KIF4A/SKA1/CENPN/KIF23/PRC1/TRIP13/DSCC1/SKA3/CENPE/CENPF/SPC25/RMI2/ZWINT/NDC80/FAM83D/NCAPG/BUB1/TOP2A/CCNB1/NEK2/NUF2/NSMCE2/KIF18A/KIF14/TTL/TTK/NUSAP1/RAD51C/MAD2L1/RACGAP1/SMC4/DLGAP5/ECT2/SPDL1/NUP37/KNSTRN/CENPQ
BP	GO:0000280	nuclear division	<0.0001	ACTR3/TPR/RAD21/RAN/PLCB1/SMC1A/FANCD2/MKI67/PKMYT1/AURKA/CDCA5/UBE2C/H2AFY/CDC20/PLK1/CKS2/MAD2L1BP/DMC1/HSPA2/KIF2C/INCENP/CDC25C/CDC6/KIFC1/CDCA8/CHEK1/BUB1B/NCAPH/KIF4A/KIF23/TPX2/PRC1/TRIP13/DSCC1/CENPE/KIF11/KNTC1/BRCA2/CENPF/ZWINT/NDC80/NCAPG/CHEK2/TOP2A/CCNB1/NEK2/ANLN/NSMCE2/KIF18A/ASPM/KIF14/TTK/NUSAP1/RAD51C/MAD2L1/RACGAP1/CALR/SMC4/DLGAP5/MTBP/SPDL1/PSMC3IP/FIGNL1/TDRKH/KNSTRN/KIF2A
BP	GO:0048285	organelle fission	<0.0001	ACTR3/TPR/RAD21/RAN/PLCB1/SMC1A/FANCD2/MKI67/PKMYT1/AURKA/CDCA5/UBE2C/H2AFY/CDC20/PLK1/CKS2/MAD2L1BP/DMC1/HSPA2/KIF2C/INCENP/CDC25C/CDC6/KIFC1/CDCA8/CHEK1/BUB1B/NCAPH/KIF4A/MAPT/KIF23/TPX2/PRC1/TRIP13/MTFR2/DSCC1/CENPE/KIF11/KNTC1/BRCA2/CENPF/ZWINT/NDC80/NCAPG/CHEK2/TOP2A/CCNB1/NEK2/ANLN/NSMCE2/KIF18A/ASPM/KIF14/TTK/NUSAP1/RAD51C/MAD2L1/RACGAP1/CALR/SMC4/DLGAP5/MTBP/SPDL1/PSMC3IP/FIGNL1/TDRKH/KNSTRN/KIF2A
CC	GO:0098687	chromosomal region	<0.0001	TPR/RAD21/MCM5/PURA/EZH2/SMC1A/PCNA/MSH2/CBX1/PARP1/DSN1/CDCA5/BIRC5/XRCC1/H2AFY/PLK1/RAD51/DMC1/KIF2C/INCENP/MCM2/DNMT1/SUV39H1/CENPW/FEN1/HJURP/CDCA8/CHEK1/BUB1B/CENPA/SKA1/CENPN/HELLS/MCM4/DSCC1/SKA3/CENPE/MCM3/CENPH/KNTC1/BRCA2/CENPF/SPC25/ZWINT/NDC80/NCAPG/CHEK2/OIP5/BUB1/CDK1/CENPI/CCNB1/NEK2/NUF2/NSMCE2/KIF18A/ITGB3BP/TTK/MAD2L1/CENPL/CENPK/MCM6/MTBP/SPDL1/NUP37/ZWILCH/KNSTRN/CENPQ

Term	ID	Description	P. adjusted	Gene
CC	GO:0000775	chromosome, centromeric region	<0.0001	TPR/RAD21/SMC1A/CBX1/DSN1/CDCA5/BIRC5/H2AFY/PLK1/KIF2C/INCENP/DNMT1/SUV39H1/CENPW/HJURP/CDCA8/BUB1B/CENPA/SKA1/CENPN/HELLS/DSCC1/SKA3/CENPE/CENPH/KNTC1/CENPF/SPC25/ZWINT/NDC80/NCAPG/BUB1/CENPI/CCNB1/NEK2/NUF2/KIF18A/ITGB3BP/TTK/MAD2L1/CENPL/CENPK/MTBP/SPDL1/NUP37/ZWILCH/KNSTRN/CENPQ
CC	GO:0000793	condensed chromosome	<0.0001	RAD21/SMC1A/DSN1/TOPBP1/MKI67/CDCA5/BIRC5/H2AFY/PLK1/RAD51/HSPA2/KIF2C/INCENP/SUV39H1/CENPW/TUBG1/HJURP/CHEK1/BUB1B/NCAPH/CENPA/SKA1/CENPN/SKA3/CENPE/CENPH/KNTC1/BRCA2/CENPF/SPC25/ZWINT/NDC80/NCAPG/BUB1/TOP2A/CCNB1/NEK2/NSMCE2/ITGB3BP/MAD2L1/HMGB2/SMC4/CENPK/NCAPG2/SPDL1/NUP37/ZWILCH/KNSTRN
MF	GO:0008017	microtubule binding	0.0002	MAP2/GAS8/BIRC5/LZTS1/PLK1/KIF2C/SKA1/MAPT/KIF23/PRC1/FGF13/RAE1/CENPE/FAM83D/APC2/GAS2L3/KIF18A/KIF14/RACGAP1/MAPRE1/MAP4K4/KIF2A
MF	GO:0003682	chromatin binding	0.0011	VRK1/TPR/TRIM24/SMAD2/MORC2/RAN/SMARCC1/EZH2/SMC1A/PCNA/MSH2/CBX1/GMNN/PPARG/BMI1/CDCA5/STAT1/H2AFY/CBX2/CKS2/RAD51/SMARCD1/DNMT1/SUV39H1/PRKAA2/CENPA/CHAF1B/UBE2T/CENPF/ZKSCAN3/CDK1/TOP2A/H2AFZ/EXO1/MCM8/POLA1/HMGN4
MF	GO:0015631	tubulin binding	0.002	TPR/MAP2/GAS8/BIRC5/LZTS1/PLK1/KIF2C/SKA1/MAPT/KIF23/PRC1/FGF13/RAE1/CENPE/BRCA2/FAM83D/APC2/GAS2L3/KIF18A/KIF14/RACGAP1/MAPRE1/CCT5/MAP4K4/KIF2A

Table S4: Result of KEGG pathway enrichment analysis based on the upregulated genes co-expressed with *KTN1* in hepatocellular carcinoma. KEGG, Kyoto Encyclopedia of Genes and Genomes.

**ID**	**description**	**P**	**FDR**	**Gene**
hsa04110	Cell cycle	<0.0001	<0.0001	RAD21/SMAD2/MCM5/SMC1A/PCNA/PKMYT1/CDC20/PLK1/PTTG1/MCM2/CDC25C/CDC6/YWHAH/DBF4/CHEK1/BUB1B/MCM4/E2F3/MCM3/CDC45/CCNB2/CHEK2/CCNA2/BUB1/CDK1/CCNB1/CDC7/TTK/CDKN2C/CCNE2/MAD2L1/MCM6/YWHAZ
hsa03030	DNA replication	<0.0001	<0.0001	POLE2/MCM5/PCNA/POLA2/RNASEH2A/MCM2/FEN1/MCM4/MCM3/RFC4/POLA1/MCM6/PRIM1/PRIM2/RFC3
hsa04114	Oocyte meiosis	<0.0001	0.0009	SMC1A/MAPK1/RPS6KA3/PKMYT1/AURKA/FBXO43/CDC20/PLK1/PTTG1/CDC25C/YWHAH/CCNB2/BUB1/CDK1/CCNB1/CCNE2/MAD2L1/YWHAZ
hsa05206	MicroRNAs in cancer	0.0001	0.0071	SHC1/GRB2/EZH2/NRAS/MAPK1/BMI1/CDCA5/SOX4/ST14/MARCKS/DNMT1/CDC25C/KIF23/E2F3/APC2/GLS/TPM1/CCNE2
hsa04914	Progesterone-mediated oocyte maturation	0.0001	0.0071	MAPK1/RPS6KA3/PKMYT1/AURKA/PLK1/CDC25C/HSP90AB1/PIK3R3/CCNB2/CCNA2/BUB1/CDK1/CCNB1/MAD2L1
hsa03460	Fanconi anemia pathway	0.0005	0.0297	FANCD2/FANCB/EME1/RAD51/FANCI/UBE2T/BRCA2/RMI2/RAD51C
hsa03050	Proteasome	0.0007	0.0331	PSMD1/PSMD14/PSMD11/PSMB4/PSMB3/PSMD2/PSMD4/PSME3

Table S5: Result of disease ontology enrichment analysis based on the upregulated genes co-expressed with *KTN1* in hepatocellular carcinoma.

ID	Description	P	FDR	Gene
PA443455	Ataxia Telangiectasia	<0.0001	<0.0001	TIPRL/MCM5/SMC1A/PCNA/MSH2/PARP1/TOPBP1/FANCD2/TIMELESS/XRCC1/PLK1/RAD51/RRM2/MICB/MCM2/CDC25C/CDC6/DBF4/CHEK1/FANCI/MCM4/MCM3/RHNO1/CDC45/BRCA2/CHEK2/CDK1/TOP2A/EXO1/CDC7/MCM8/MCM6/WDHD1
PA445538	Retinoblastoma	<0.0001	<0.0001	CEBPA/KPNA7/MCM5/PURA/SMARCC1/EZH2/PTPN12/PCNA/PSMD10/PHB/BMI1/MKI67/BRMS1/DNMT1/SUV39H1/CDC6/CENPA/E2F8/MCM4/E2F3/PIK3R3/CCNA2/CDK1/APC2/CCNB1/CDKN3/KIF14/CDKN2C/PTK2/CCNE2/MCM6
PA443358	Aneuploidy	<0.0001	<0.0001	RAD21/SMC1A/STIL/AURKA/UBE2C/CDC20/PLK1/MAD2L1BP/PTTG1/KIF2C/INCENP/TUBG1/KIFC1/CHEK1/BUB1B/CENPA/SGCE/TPX2/MCM4/PLK4/CENPE/CENPH/KIF11/ZWINT/NDC80/BUB1/CDK1/TOP2A/NEK2/TTK/MAD2L1/MCM6
PA444146	Fanconi Anemia	<0.0001	<0.0001	MCM5/PCNA/MSH2/PARP1/TOPBP1/FANCD2/FANCB/XRCC2/STAT1/EME1/RAD51/MCM2/FEN1/CHEK1/FANCI/MCM3/UBE2T/BRCA2/RMI2/CHEK2/RFC4/RAD51C/RAD51AP1
PA443527	Bloom Syndrome	<0.0001	<0.0001	MSH2/PARP1/TOPBP1/FANCD2/RAD51/FEN1/CHEK1/RMI2/CHEK2/TOP2A/EXO1/NSMCE2/TTK/RAD51C
PA443560	Breast Neoplasms	<0.0001	<0.0001	AKR1C3/EZH2/PARP1/PHB/BMI1/FANCD2/MKI67/AURKA/BIRC5/BRMS1/XRCC2/XRCC1/APOBEC3B/LZTS1/RAD51/ST14/PTTG1/FAP/DNMT1/LAMP3/CAV1/STXBP4/GPNMB/BRCA2/CHEK2/MELK/TOP2A/LAPTM4B/CCNB1/NEK2/KIF14/TACC2/PTK2/RAD51C/CCNE2/ROBO1/HMMR/PEA15/ATAD2/ENAH/ITGA6
PA443454	Ataxia	<0.0001	0.0002	SMC1A/PARP1/TOPBP1/FANCD2/CSTB/TIMELESS/XRCC1/PLK1/RAD51/NDUFA2/MCM2/SLC1A3/CDC25C/CDC6/ABHD12/DBF4/CHEK1/FANCI/MCM4/MCM3/FLVCR1/RHNO1/CDC45/CHEK2/LMNB1/CDK1/EXO1/CDC7/MCM8/ANO10/WDHD1/DARS2
PA444447	Carcinoma, Hepatocellular	<0.0001	0.0020	ASPH/HTATIP2/CEBPA/EZH2/TP53BP2/PSMD10/BMI1/THY1/BIRC5/XRCC1/SOX4/CKS2/DNMT1/UBD/SND1/SULF1/GLUL/E2F3/ANXA2/LAPTM4B/NEK2/CDKN3/PTK2/DLGAP5/ATAD2/CAP2

ID	Description	P	FDR	Gene
PA446646	Carcinoma, Ductal, Breast	<0.0001	0.0138	ZBTB33/EZH2/GMNN/MKI67/BIRC5/BRMS1/PTTG1/FAP/BRCA2/CHEK2/TOP2A/NEK2/ENAH
PA445062	Neoplasms	<0.0001	0.0138	HTATIP2/CEBPA/AKR1C3/RRM1/EZH2/NRAS/ITGAV/MSH2/PSMD10/PARP1/BMI1/MKI67/AURKA/THY1/BIRC5/BRMS1/XRCC2/XRCC1/SOX4/CD34/LZTS1/RAD51/RRM2/TM4SF1/ST14/TNFRSF12A/PTTG1/LGALS3/FAP/PLCE1/LEF1/DNMT1/CHEK1/GRPR/SULF1/CD200/CAV1/TPX2/E2F3/GPNMB/BRCA2/CHEK2/ADAM9/ANXA2/MELK/TOP2A/LAPTM4B/CDKN3/CDKN2C/PTK2/RAD51C/CCNE2/HMMR/ATAD2/ITGA6

Table S6: Result of Wikipathway cancer enrichment analysis based on the upregulated genes co-expressed with *KTN1* in hepatocellular carcinoma.

ID	Description	P	FDR	Gene
WP2446	Retinoblastoma Gene in Cancer	<0.0001	<0.0001	HLTF/POLE2/RRM1/SMC1A/PCNA/DCK/RRM2/DNMT1/SUV39H1/CHEK1/KIF4A/MCM4/PLK4/E2F3/MCM3/CDC45/CCNB2/CCNA2/CDK1/TOP2A/CCNB1/ANLN/H2AFZ/CDC7/RABIF/RFC4/TTK/POLA1/CCNE2/HMGB2/MCM6/PRIM1/RFC3
WP179	Cell Cycle	<0.0001	<0.0001	RAD21/SMAD2/MCM5/SMC1A/PCNA/PKMYT1/CDC20/PLK1/PTTG1/MCM2/CDC25C/CDC6/YWHAH/DBF4/CHEK1/MCM4/E2F3/MCM3/CDC45/CCNB2/CHEK2/CCNA2/BUB1/CDK1/CCNB1/CDC7/TTK/CDKN2C/CCNE2/MCM6/YWHAZ
WP4016	DNA IR-damage and cellular response via ATR	<0.0001	<0.0001	SMC1A/PCNA/MSH2/PARP1/TOPBP1/FANCD2/PLK1/RAD51/MCM2/CDC25C/EEF1E1/FEN1/CHEK1/FANCI/CDC45/BRCA2/CHEK2/CDK1/EXO1
WP4240	Regulation of sister chromatid separation	<0.0001	<0.0001	RAD21/SMC1A/CDC20/PTTG1/BUB1B/CENPE/BUB1/MAD2L1
WP45	G1 to S cell cycle control	<0.0001	0.0001	POLE2/MCM5/PCNA/POLA2/MCM2/MCM4/E2F3/MCM3/CDC45/CDK1/CCNB1/CDKN2C/CCNE2/MCM6/PRIM1/PRIM2

Table S7: Result of Reactome pathway enrichment analysis based on the upregulated genes co-expressed with *KTN1* in hepatocellular carcinoma.

ID	Description	P. adjusted	FDR	Count
R-HSA-69620	Cell Cycle Checkpoints	<0.0001	<0.0001	71
R-HSA-68886	M Phase	<0.0001	<0.0001	71
R-HSA-68882	Mitotic Anaphase	<0.0001	<0.0001	50
R-HSA-2555396	Mitotic Metaphase and Anaphase	<0.0001	<0.0001	50
R-HSA-2500257	Resolution of Sister Chromatid Cohesion	<0.0001	<0.0001	40
R-HSA-2467813	Separation of Sister Chromatids	<0.0001	<0.0001	48
R-HSA-68877	Mitotic Prometaphase	<0.0001	<0.0001	49
R-HSA-69306	DNA Replication	<0.0001	<0.0001	37
R-HSA-141424	Amplification of signal from the kinetochores	<0.0001	<0.0001	32
R-HSA-141444	Amplification of signal from unattached kinetochores via a MAD2 inhibitory signal	<0.0001	<0.0001	32
